# Systematic Comparisons for Composition Profiles, Taxonomic Levels, and Machine Learning Methods for Microbiome-Based Disease Prediction

**DOI:** 10.3389/fmolb.2020.610845

**Published:** 2020-12-16

**Authors:** Kuncheng Song, Fred A. Wright, Yi-Hui Zhou

**Affiliations:** ^1^Bioinformatics Research Center, North Carolina State University, Raleigh, NC, United States; ^2^Departments of Statistics and Biological Sciences, North Carolina State University, Raleigh, NC, United States; ^3^Department of Biological Sciences, North Carolina State University, Raleigh, NC, United States

**Keywords:** phenotype prediction, machine learning method, k-mers, operational taxonomic unit (OTU), amplicon sequence variant (ASV), phylogenetic analysis

## Abstract

Microbiome composition profiles generated from 16S rRNA sequencing have been extensively studied for their usefulness in phenotype trait prediction, including for complex diseases such as diabetes and obesity. These microbiome compositions have typically been quantified in the form of Operational Taxonomic Unit (OTU) count matrices. However, alternate approaches such as Amplicon Sequence Variants (ASV) have been used, as well as the direct use of k-mer sequence counts. The overall effect of these different types of predictors when used in concert with various machine learning methods has been difficult to assess, due to varied combinations described in the literature. Here we provide an in-depth investigation of more than 1,000 combinations of these three clustering/counting methods, in combination with varied choices for normalization and filtering, grouping at various taxonomic levels, and the use of more than ten commonly used machine learning methods for phenotype prediction. The use of short k-mers, which have computational advantages and conceptual simplicity, is shown to be effective as a source for microbiome-based prediction. Among machine-learning approaches, tree-based methods show consistent, though modest, advantages in prediction accuracy. We describe the various advantages and disadvantages of combinations in analysis approaches, and provide general observations to serve as a useful guide for future trait-prediction explorations using microbiome data.

## Introduction

With the advancement of sequencing technology and the downward trends in the cost of sequencing, more studies have used microbiome data as a primary source for investigating the relationship between the microbiota and host health. In general, human microbiota samples consist of easily collected specimens such as feces, saliva, and skin. Upon collection, the sample can undergo a variety of extraction protocols, including from protein, RNA, and DNA. Each of these data types has led to a specific field of emerging research (Weinstock, 2012). In this review, we focus on the targeted extraction of microbial DNA from the16S rRNA region, which is present in most microorganisms but displays high variability across species. The sequenced reads are then typically clustered into Operational Taxonomic Units (OTUs) by matching the reads to a reference database.

Multiple studies have investigated the use of OTUs for phenotype/disease prediction, including inflammatory bowel diseases (Gevers et al., 2014), Type 2 diabetes (Gurung et al., 2020), and lung cancer (Zheng et al., 2020). As a variety of data treatment and prediction methods have been used, there is a pressing need to connect and verify how the upstream processing of the 16S rRNA data affects the downstream prediction performance, and compare among the different OTU/ASV methods.

There are two primary representations to produce data count matrices: OTUs and Amplicon Sequence Variants (ASVs) (Rosen et al., 2012). Within the realm of OTUs, there are three methods to “cluster” sequences into OTUS: *de novo*, closed-reference, and open-reference, each with its unique advantages and disadvantages depending on the sequence region, reference database, and sample environment (Rideout et al., 2014). ASVs are commonly generated using the Divisive Amplicon Denoising Algorithm 2 (DADA2), and the resultant ASVs represent true biological sequences obtained from reads (Callahan et al., 2016). In addition, there have been recent efforts to use the occurrence of short-chain k-mer (15–30-mer) (Molik et al., 2020), and very short-chain k-mers (<10-mer) (Asgari et al., 2018, 2019), within reads that offer a unique reference-free and alignment-free approach to provide a data representation upon which a phenotype prediction model is built. We have included both of these k-mer approaches in our review to compare them directly with the OTU/ASV assignment methods.

Additional procedures for handling the OTUs or ASVs include filtering (Goodrich et al., 2014; Duvallet et al., 2017; Zhou and Gallins, 2019) and normalization (McMurdie and Holmes, 2014; Weiss et al., 2017). We included both practices to show the result from different combinations.

Overall, we conducted a systematic review of how different combinations of (i) OTU/ASV assignment methods and k-mer lengths, (ii) the use of normalization and filtering and (iii) choices of machine learning methods, among eleven commonly used approaches, all affect the prediction accuracy for complex host traits.

## Methods

### Raw Sequence Data

#### Inflammatory Bowel Diseases Dataset

This microbiome dataset includes host phenotypes of Crohn's disease, with microbiome data from 16S rRNA gene (V4) sequencing on the Illumina MiSeq platform (version 2) with 175 bp paired-end reads (Gevers et al., 2014). In brief, the samples were collected from 28 participating pediatric gastroenterology centers in North America between 2008 and 2012. Within the metadata, there are three disease diagnoses described: Crohn's Disease (CD), Ulcerative Colitis (UC), Ischemic Colitis (IC), and control. Each of the disease diagnoses was compared separately to the control group. The data were downloaded from the European Nucleotide Archive (ENA), accession PRJEB13679. The available FASTQ file format is a single-end layout; the QIIME2 pipeline for the microbiota analyses was processed as single-end reads. The full processing workflow is described in the Supplementary Material under Data Processing and Supplementary Figure 1. A summary of the basic patient characteristics for the datasets is provided in Table 1.

**Table 1:** Brief summary of datasets.

	**Study**	**Inflammatory Bowel Diseases**				**Twins UK**	
	**Disease type**	**Crohn's disease**	**Ischemic colitis**	**Ulcerative colitis**	**Control**	**Obesity**	**Healthy**
	*n*	731	73	217	335	193	451
Sex	Female	337	38	96	161	192	447
	Male	394	35	123	174	1	4
Bases per FASTQ file	Mean	6,381,116	6,414,195	6,884,041	7,247,420	19,776,834	20,355,603
	*SD*	7,184,600	5,026,312	5,857,603	8,069,537	5,421,961	6,004,283
Sequence length	Mean	172.44	172.52	172.52	172.99	250.84	250.84
	*SD*	1.37	1.18	1.36	1.12	0.40	0.41
Age	Mean	19.92	18.15	26.93	13.78	60.49	59.84
	*SD*	14.47	10.77	18.31	9.78	9.56	9.57

#### TwinsUK Dataset

This microbiome dataset contains 1,081 fecal samples collected from 997 individuals, all of which underwent 16S rRNA-based sequencing. The raw sequences were retrieved from the European Nucleotide Achieve (ENA) accession IDs PRJEB6702 and PRJEB6705. The collection and processing of the data were described previously (Goodrich et al., 2014). The fecal samples were obtained by the participants from their household and stored in a refrigerator up to 2 days prior to the twins' annual visit at King's College London, where the samples were stored at −80C until the following process. The DNA was extracted from the provided samples, and the 16S rRNA genes (V4) were amplified from bulk DNA through PCR. The sequencing steps were performed on the Illumina MiSeq 2x250 bp platform. The available FASTQ file format is a single-end layout; the QIIME2 pipeline for the microbiota analyses was processed as single-end reads. The full processing is described in Supplementary Section 1 under Data Processing. The summary of the basic patient characteristics is included in Table 1.

### Sample Processing

**Figure 1:**
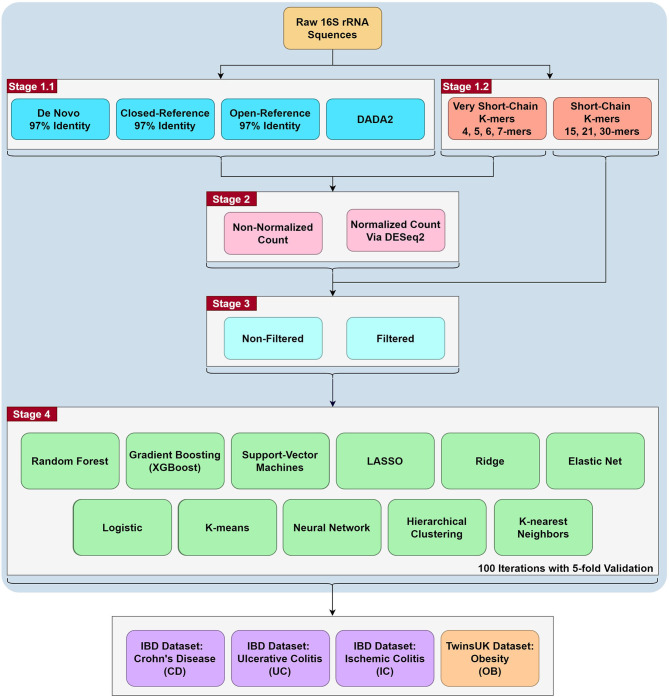
The workflow of the project. The project is roughly split to four stages. The first stage is the generation of count matrices via QIIME2 for the OTU/ASV assignment methods, while the k-mer matrices were generated using R (resulting in 35 count matrices). In the second stage, DESeq2 normalization are performed for all stage 1 count matrices except for the short-chain k-mers (resulting in 67 count matrices). In the third stage, filtering was performed for all the above count matrices (resulting in 123 count matrices). In the fourth stage, we ran eleven commonly used machine learning methods on the 123 count matrices with 100 iterations of 5-fold validation (resulting in 1,353 combinations). Lastly, we tested these combinations with 4 binary comparisons: Crohn's Disease, Ulcerative Colitis, Ischemic Colitis and Obesity with the corresponding control in their respective dataset (resulting in 5,412 combinations). A more detailed workflow is in Supplementary Figure 1.

The detailed sample processing is also listed in the Data Processing Supplementary Section 1, and the workflow is shown in Figure 1. Our analyses can be summarized in four stages. In the first stage, we extracted the OTUs/ASVs using QIIME2 (Bolyen et al., 2019). We then collapsed count matrices at OTUs/ASVs levels to higher taxonomic order, including phylum, class, order, family, genus, and species. At the same time, we also extracted the very short-chain k-mers and short-chain k-mers directly from raw FASTQ files. In the second stage, we used the DESeq2 package in R to apply normalization to the OTUs/ASVs count matrices and the very short-chain k-mers (Weiss et al., 2017), or did not apply normalization. Short-chain k-mer (15-mer, 21-mer, and 30-mer) were omitted from this analysis because of the large matrix dimensions when including all observed short k-mers. In the third stage, we applied (or did not) a common filtering criterion as follows. The first filter excludes samples with fewer than 100 reads, and the second filter subtracts OTUs with fewer than 10 reads (Duvallet et al., 2017; Zhou and Gallins, 2019). The third filter removes OTUs that are present in fewer than 5% of samples. In the last stage, we applied eleven commonly used machine learning algorithm to the different combinations. Overall, we conducted 1,353 combinations per phenotype and 5,412 total combinations for four diseases against their respective controls.

In our extensive analyses, 102 combinations failed to return any useful results. Thirteen of these involved elastic nets, five used neural networks, and the remaining 84 combinations used logistic regression. These failed runs were likely due to the algorithms being unable to converge.

### Evaluation of Prediction Accuracy

We compared the four disease types prediction at each taxonomic and k-mer level through the Area Under the Curve (AUC) for the Receiver Operating Characteristics (ROC) curve, which is commonly used to evaluate the prediction accuracy for binary traits. The ROC is a plot with True Positive Rate (Sensitivity) compared to the False Positive Rate (1 – Specificity). Also, we can calculate the Area Under the Precision-Recall Curve (AUPR), which is another way to evaluate the prediction with a plot of recall against precision. We utilized two evaluating parameters to quantify the prediction ability of a balanced and imbalanced dataset. Ideally, we would want these two values to be both high to indicate good discrimination between the disease and the controls. The full summary of the combinations is in Supplementary Table 1. In the following discussion, we focus primarily on the AUC (full abbreviation AUROC for Area Under the ROC) as a performance measure, as it offers a more distinct contrast among combinations compared to the AUPR for our comparisons.

### Evaluation of Machine Learning Results

To investigate the consistency between the feature selected from machine learning algorithms and the discovery studies, we extracted the useful information from the machine learning algorithm outputs and compared them to taxa previously identified as significantly associated with IBD. We based our comparisons on three separate publications. First, we selected eleven critical taxa identified from the original study for our IBD dataset (Gevers et al., 2014): two from the Order level and nine from the Family level. All of these were identified in our results, with the exception of a Family-level assignment *Gemellaceae*, and we used the Genus-level assignment *Gemella* as a substitute. Also, we chose another study that had examined the microbiota associated with IBD; the authors had identified multiple taxa associated with either increased or decreased changes in IBD (Glassner et al., 2020), and we selected nine taxa from the list. The Genus *Bacteroides* and *Eubacterium* have multiple subgroups, and the *Pectinophilus* group was selected for *Bacteroides*, and the *nodatum* group was selected as a stand-in for *Eubacterium*, and both passed our filtering procedures. In the last example, the authors had used a linear discriminant analysis effect size approach to determine three important taxa, two from the Family-level and one from the Order level, all of which are present in our features (Kim et al., 2019).

We focused on two of the most consistent machine learning methods, random forest and xgBoost, and two methods with less consistent performance, a support vector machine and logistic regression. The definition of “important” features is different depending on the method. Each of the features was selected within the 100 iterations of 5-fold cross-validation. As a result, the numeric representation of “important” features here represents an average over 500 training and testing loops.

The results on presence of taxa are shown in Table 2. Overall, only DADA2 was able to pick all these taxa while other OTU assignment methods missed a few. After filtering, most of the taxa listed were removed; out of the 22 taxa, only nine taxa remained, and these nine taxa were present in all OTU/ASV assignment methods, except *Eubacterium* with the *nodatum* group that were missing when using the *de novo* method.

**Table 2:** Presence of important taxa in our clustering methods.

**Gevers et al**.	**Glassner et al**.	**Kim et al**.	**Taxonomic Level**	**Open-Reference**	**Closed-Reference**	***de novo***	**DADA2**
Bacteroidales			Order				Present
Clostridiales			Order	Present	Present	Present	Present
		Lactobacillales	Order				Present
*Coriobacteriaceae*			Family	Present	Present	Present	Present
*Enterobacteriaceae*			Family	Present	Present	Present	Present
*Erysipelotrichaceae*			Family	Present	Present	Present	Present
Fusobacteriaceae^*^			Family	Present	Present	Present	Present
Micrococcaceae^*^			Family	Present	Present	Present	Present
*Neisseriaceae*			Family	Present	Present	Present	Present
*Pasteurellaceae*	*Pasteurellaceae*		Family	Present	Present	Present	Present
*Veillonellaceae*	*Veillonellaceae*		Family	Present	Present	Present	Present
Verrucomicrobiaceae^*^			Family	Present	Present	Present	Present
		Pseudomonadaceae^*^	Family	Present	Present	Present	Present
		Streptococcaceae^*^	Family	Present	Present	Present	Present
*Gemella*			Genus	Present	Present		Present
	Bacteroides^*^^‡^		Genus	Present	Present	Present	Present
	*Bifidobacterium*		Genus				Present
	Eubacterium^*^^†^		Genus	Present	Present		Present
	Faecalibacterium^*^		Genus	Present	Present	Present	Present
	*Fusobacterium*		Genus	Present	Present	Present	Present
	Roseburia^*^		Genus	Present	Present	Present	Present
	*Solobacterium*		Genus	Present	Present	Present	Present

* *The Taxa that were kept after filtering was performed*.

‡ *There are multiple groups under Bacteroides, Pectinophilus group was selected as it is the only Eubacterium group that remains after our filtering procedure*.

† *There are multiple groups under Eubacterium - the nodatum group was selected as it is the only Eubacterium group that remains after our filtering procedure*.

*Present: the taxa occurred in the features from the corresponding non-filtered OTU/ASV assignment method*.

*An empty cell means the taxon is absent in the corresponding OTU/ASV assignment method*.

#### Defining Important Features

There are many ways to define the features from machine learning models that are important to the model. For illustrative purposes, we will focus on only one of the available ways to select important features. With xgBoost, we extracted the “Gain” result from the xgBoost output to evaluate the importance of the features (Chen and Guestrin, 2016). Gain represents the relative contribution of the corresponding feature to the model based on each tree in the training data. In other words, the higher the Gain, the more critical that feature is compared to other features. For random forests, we selected the “Mean Decrease in Gini” output to evaluate the importance of features (Breiman, 2001). Mean Decrease in Gini represents how each feature contributes to the homogeneity of the nodes and leaves in the given random forest model. Hence, the higher the Mean Decrease Gini, the more critical the corresponding feature. We utilize the weights associated with each of the features to evaluate their importance in the support vector machine (Chih-Chung Chang, 2019). These weights represent the feature's discriminative ability to distinguish between two classes: the higher the weights, the more crucial the support vector machine model's feature. Lastly, in logistic regression, we obtained the coefficients from each of the iterations, and then checked the consistency of the coefficients across multiple iterations.

### Evaluation of Phylogeney-Aware Distances

Phylogeny-aware distances are used to determine if we can separate species between different communities in an aggregate fashion. In our analyses, we examined the distances using multiple types of distances, including Euclidean (Schloss, 2008), Jaccard (Hancock and Zvelebil, 2004), Bray-Curtis (Bray and Curtis, 1957), UniFrac (Lozupone and Knight, 2005), and weighted UniFrac (Lozupone et al., 2007). Euclidean distance is a traditional distance measure between two species. The Jaccard index is a similarity coefficient using the presence and absence of the features within the OTU/ASV matrices. The Bray-Curtis distance is a widely used technique to highlight the differences in abundance by transforming the count matrix to a distance matrix. UniFrac, in contrast, utilizes the phylogenetic tree structure and its distances to calculate the overall distance matrix. Weight UniFrac takes account of the relative abundance of information and weights the branches of the phylogenetic tree.

## Results

### Prediction Accuracy

#### OTU/ASV Assignment Methods

**Figure 2:**
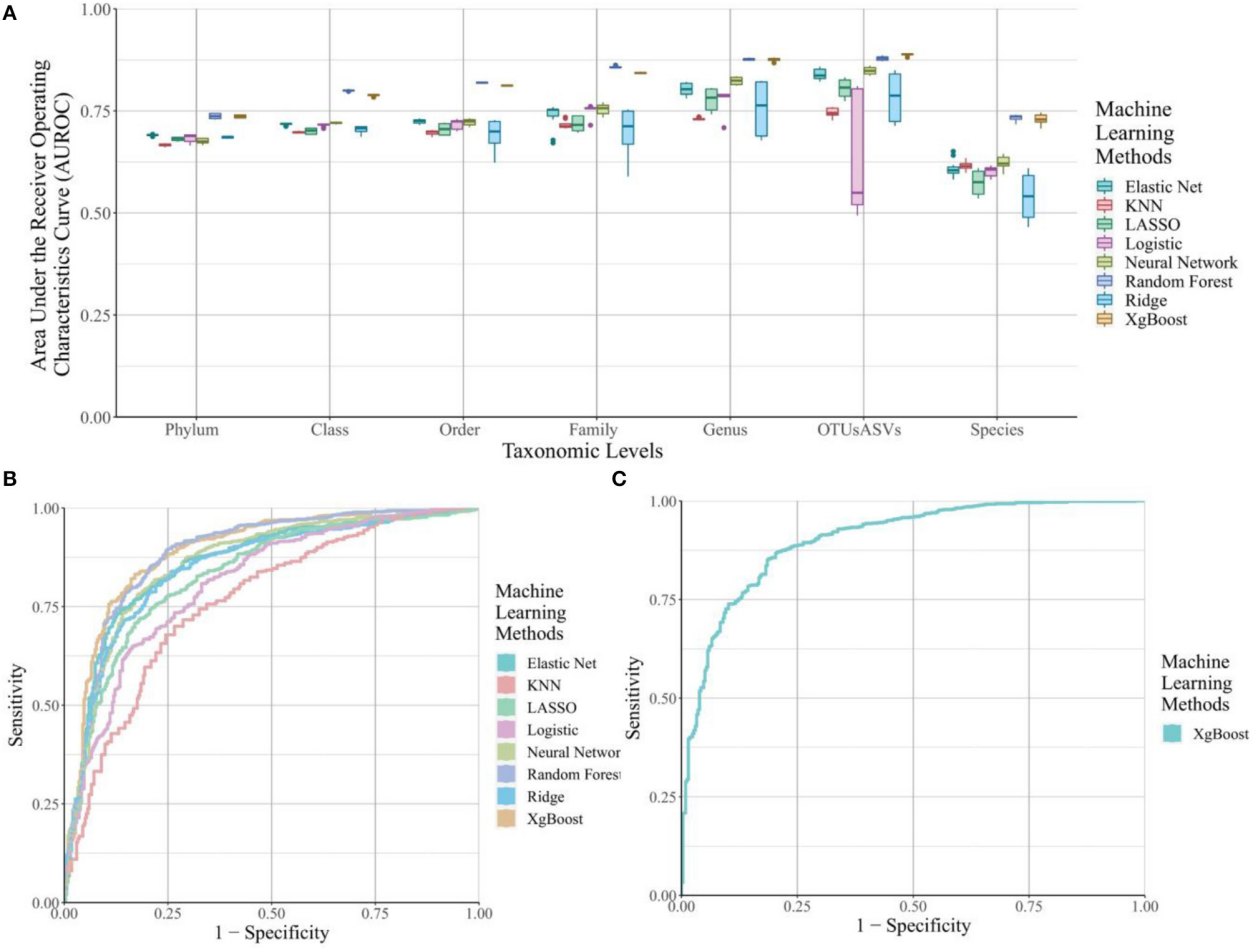
The area under the ROC curve (AUROC) for selected machine learning methods across different taxonomic levels and k-mer lengths. **(A)** Boxplots of the AUROC for eight machine learning methods from OTU/ASV assignment methods across all seven taxonomic levels for Crohn's Disease. **(B)** ROC curves for the eight machine learning methods from OTU/ASV assignment methods across all seven taxonomic levels. Hierarchical clustering, K-means and Support Vector Machine were removed from the figure due to their poor performance. **(C)** ROC curve for the best k-mer methods to predict Crohn's Disease, which is from the xgBoost algorithms on the 7-mer level.

For the traditionally-used OTUs and ASVs count matrices (Figure 2A), the prediction accuracy was lower at higher taxonomic levels, such as Phylum and Class, and gradually increased for most machine learning methods until reaching the OTU/ASV level of refinement. The highest average prediction accuracies are at the Genus and OTU/ASV level. This observation provides support for the common use of this level of taxonomy in phenotype prediction. All machine learning algorithms with an average around or below 0.5 were dropped in the figure, because those algorithms do not assist in distinguishing cases and controls. This step excludes support vector machines, K-means, and hierarchical clustering.

The noticeable drop at prediction accuracy with Species-level information is due to incomplete information in the taxonomic assignment of the reference database. As a result, these missing assignments were dropped before running the machine learning algorithms, resulting in decreased performance. The number of unique feature counts for each of the taxonomic level are listed in Supplementary Table 2. Overall, the number of unique features for the Species-level was ~half that of the Genus level in the unfiltered category. After filtering, the number of unique features is close to the Order-level or Family-level information, explaining the drop we observed in Figure 2A.

We also extracted the top-performing combination and its associated ROC curve in Figure 2B; the tree-based methods, random forests and xgBoost, performed the best, followed by neural networks, elastic net, ridge regression, LASSO regression, logistic regression, and KNN. The AUROCs for all of these methods are above 0.8.

**Figure 3:**
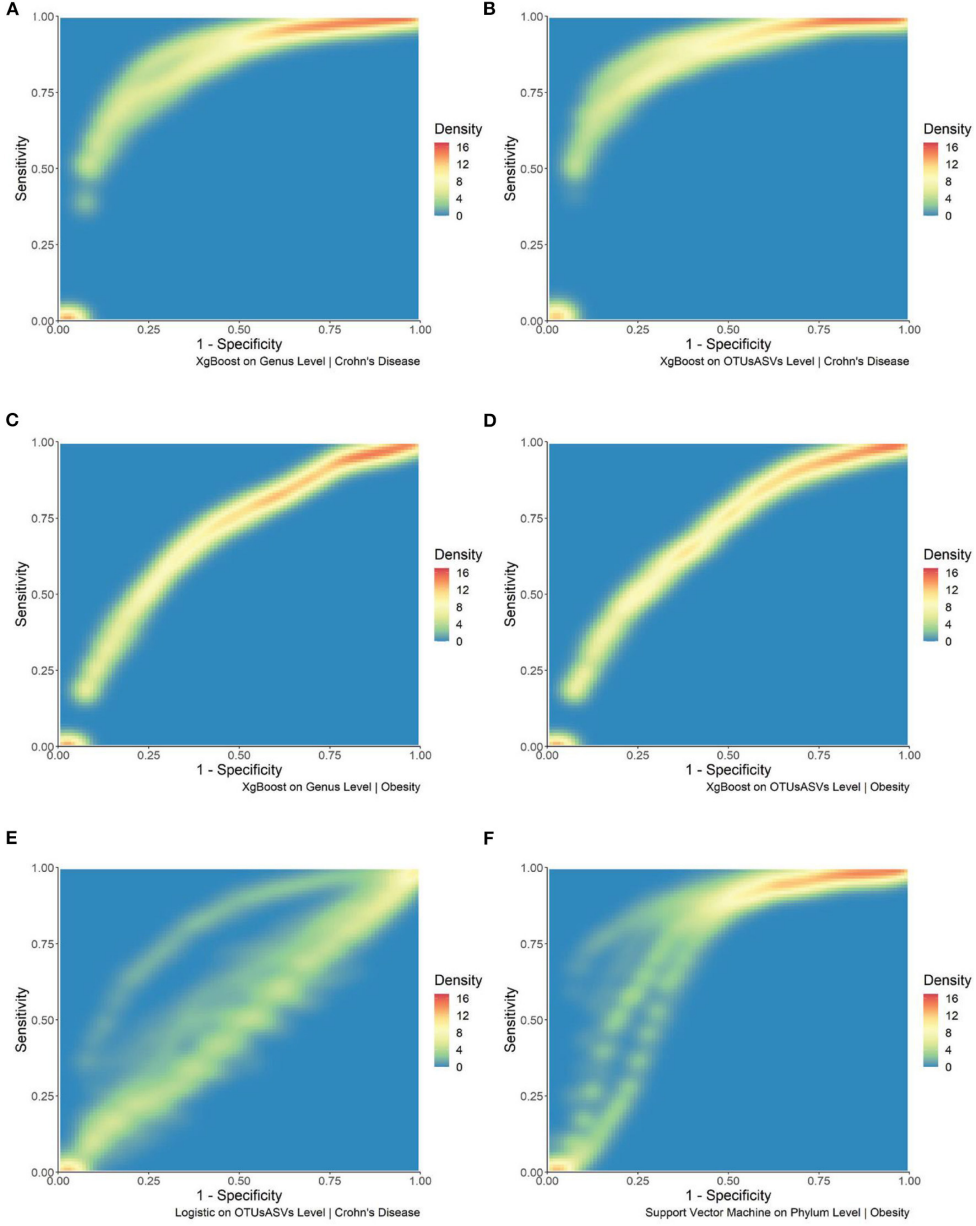
Density plots of selected combination of machine learning methods, taxonomic levels, and dataset. **(A)** Density plot of the ROC curve for xgBoost at the Genus level for the IBD dataset. **(B)** Density plot of the ROC curve for xgBoost at the OTU/ASV level for the IBD dataset. **(C)** Density plot of the ROC curve for xgBoost at Genus level for the TwinsUK dataset. **(D)** Density plot of the ROC curve for xgBoost at the OTU/ASV level for the TwinsUK dataset. **(E)** Density plot of the ROC curve for logistic regression at the OTU/ASV level for the IBD dataset. **(F)** Density plot of the ROC curve for support vector machines at the Phylum level for the TwinsUK dataset.

To further investigate different machine learning algorithms' performance, we investigated a single machine learning algorithms' performance for each disease type at a single taxonomic level. In Figures 3A–D, we observed in density plots for ROC curves the consistent xgBoost performance at the Genus and OTU/ASV levels for both diseases. Each of the plots reflects sixteen different combinations from four OTU/ASV assignment methods, two filtering, and two normalization methods. XgBoost is consistent in its performance under different combinations. As a contrast, we also included two inconsistent results. In Figure 3E, we showed the logistic regression for the inflammatory bowel disease (IBD) dataset at the OTU/ASV level; we observed some excellent performing combinations and a cluster of mediocre ROC curves. Another example came from the Phylum-level support vector machine from the TwinsUK dataset shown in Figure 3F. This density plot contains two of the best performing combinations in our entire set of 5,412 combinations. The combinations used DADA2 both with filtered features; the non-normalized and normalized AUCs are 0.8977 and 0.8965, respectively. However, we also observed the other combinations from different OTU/ASV assignment methods perform less well.

#### k-mer-Based Methods

We examined prediction accuracy of different k-mers, and no clear trend was observed; the prediction accuracy is relatively consistent across all lengths (not shown) thus, we display only the top-performing method (Figure 2C), which is the xgBoost combination using 7-mers for predicting Crohn's Disease. This combination has an AUROC of 0.924. The breakdown of AUROC per disease type at different k-mer lengths can be observed in Supplementary Figures 4–7 for Crohn's Disease, Interstitial Cystitis, Obesity and Ulcerative Colitis, respectively.

### Filtered vs. Unfiltered

**Figure 4:**
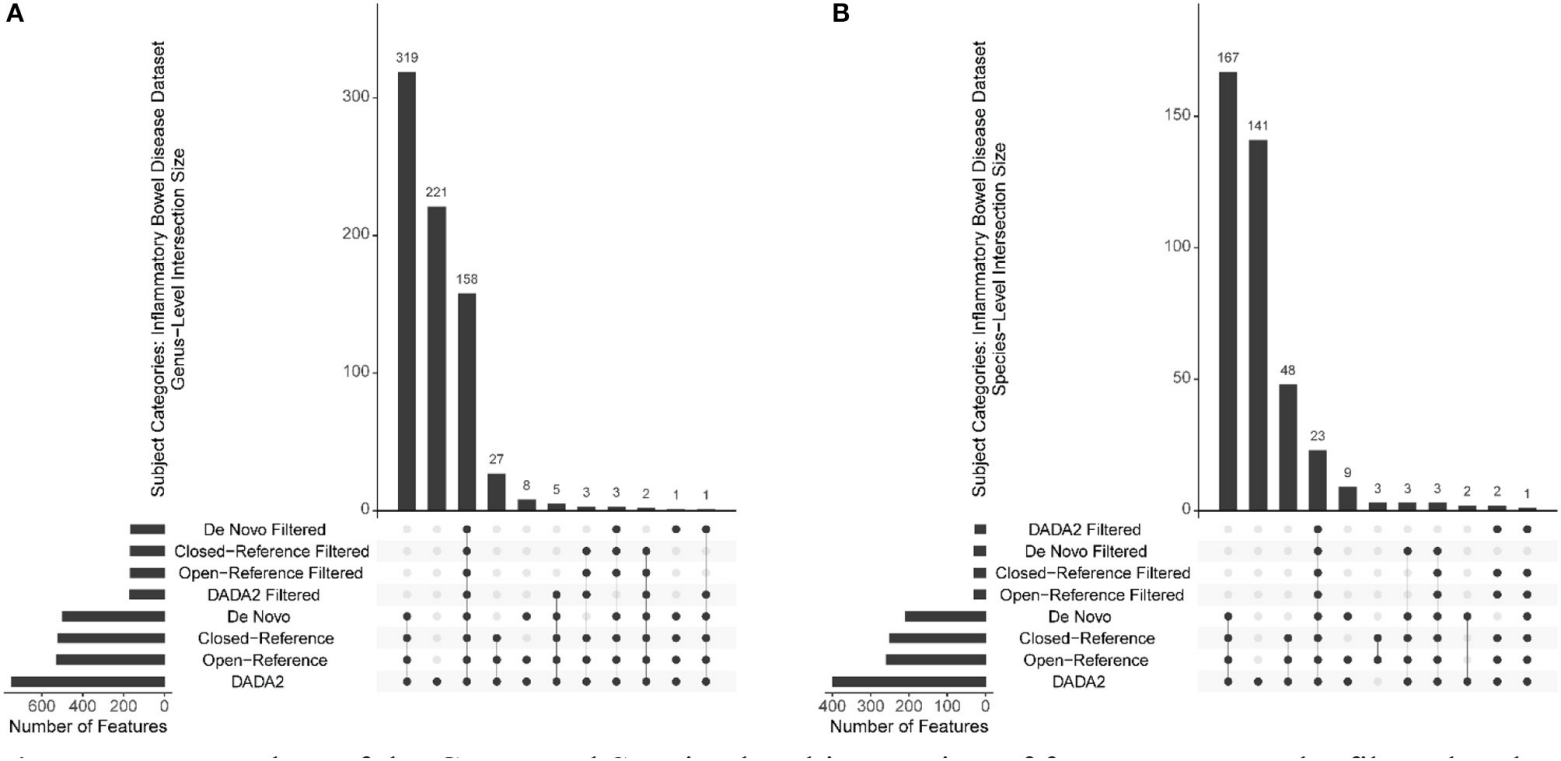
Upset plots of the Genus and Species-level interaction of features among the filtered and non-filtered OTU/ASV picking methods from the Inflammatory Bowel Disease Dataset. **(A)** Genus-level. **(B)** Species-level.

To investigate how the filtering affects the final features selected from different OTU/ASV assignment methods at different taxonomic levels and to compare these methods, we utilized “UpSet” plots to show the unique taxa shared among filtered and non-filtered methods. Regardless of filtering, the filtered and unfiltered four OTU/ASV assignment methods provide very similar unique features at the Phylum, Class, Order, and Family-level. As expected, there are more unique features identified for the Genus and Species level. In Figures 4A,B, we provided examples from the Genus and Species-level for the Inflammatory Bowel Disease dataset (with expanded plots for other diseases in Supplementary Figures 2, 3). While most of them are shared, each of the OTU/ASV identified different sets of unique features, which might hold keys to better prediction and are important for future investigations. The number of features found per taxonomic level for each of the sub-disease categories is included in Supplementary Table 1.

Filtering, in general, did not cause a severe difference in terms of AUROC for most of the machine learning methods. The exception is Logistic regression and K-means. Filtering improves the prediction accuracy in Logistic Regression in Family, Genus, and Species levels for both of normalization categories (Supplementary Figure 8). However, the results are not consistent, and thus, filtering needs to be judged case-by-case. Supplementary Table 2 provides the AUROC and AUPR for all prediction combinations and disease used in this study. The AUROC is more inconsistent at more precise taxonomic levels due to removal of features as we refine the taxonomic assignments.

### Consistency

Consistency is a key feature when investigating different prediction methods, as we have shown that some machine learning methods might be sensitive to a particular OTU/ASV assignment method. The detailed breakdown for each disease type is included in Supplementary Figures 4–7 for Crohn's Disease, Interstitial Cystitis, Obesity, and Ulcerative Colitis, respectively.

We also investigated the change in prediction accuracy in terms of AUROC by filtering and normalization individually. When we compared the difference between the filtered vs. unfiltered (Supplementary Figure 8) and normalized vs. un-normalized (Supplementary Figure 9) across the disease categories. Overall, the difference in terms of AUROC are fairly small for most of the machine learning methods with the exception of K-means and Logistic Regression. Filtering seems to cause more instability of the AUROC as the difference are more obvious. Overall, the decision of using normalization and filtering should be evaluated by the data in-hand and the purpose of the study.

### Shiny App

Considering the vast number of combinations we have tested, and to help to visualize and understand the different types of combinations we have generated, we have deployed an Shiny application: https://github.com/zhouLabNCSU/MicrobiomePredictionExplorer.

### Compare Machine Learning Methods to Discovery Studies

#### Difference Among OTU/ASV Assignment Methods

The feature selection outputs from the four different machine learning methods are consistent within the filtered and unfiltered combination for all OTU/ASV assignment methods. The top-ranked features from both random forest and xgBoost were mostly features that had passed our filtering protocol. The support vector machine approach had a less consistent output; the rankings were similar only within the filtered and unfiltered categories. The ranks from the support vector machine were also quite different compared to xgBoost and random forest. The consistency of the coefficients is also a crucial tool to understand the properties of a good predictor for logistic regression.

To better understand the feature output, we ranked the output, and the findings are shown in the Supplementary Table 3. Based on the preliminary findings, there is no noticeable difference between the normalized and un-normalized combinations under the same filtering and OTU/ASV assignment methods. Thus, the ranks we shared in Supplementary Table 3 contain only the unnormalized dataset.

#### Features Selected

The three order-level taxa all displayed average or below-average rankings. XgBoost excluded all of these taxa, as they did not help with prediction. For the eleven family-level features, the five taxa that passed the filtering procedure, *Fusobacteriaceae, Micrococcaceae, Verrucomicrobiaceae, Pseudomonadaceae, and Streptococcaceae* were all ranked around the average, with none of them performing very well. Random forests, xgBoost, and support vector machines shared similar results. Lastly, among the eight genus-level taxa, *Bacteroides* (*Pectinophilus* group) and *Roseburia* ranked among the top 10 for the random forests, xgBoost, and support vector machines with consistent results in logistic regression. The exception is *Roseburia* in support vector machines, which ranked much higher.

Overall, the rankings for random forests and xgBoost were similar between the filtered and unfiltered combinations across all four OTU/ASV assignment methods. In other words, the excess taxa unique to the unfiltered dataset did not improve the prediction accuracy, as the ranks did not change much even after adding a large number of features to the model. However, in the support vector machine, the taxa ranks were inconsistent between the filtered and unfiltered OTU/ASV assignment methods. The ranks remain roughly around the same percentile.

### Phylogenetic Analyses

#### Differential Taxa in Crohn's Disease

Overall, the weighted UniFrac was the best performing way to separate the Crohn's Disease and Control subjects.

**Figure 5:**
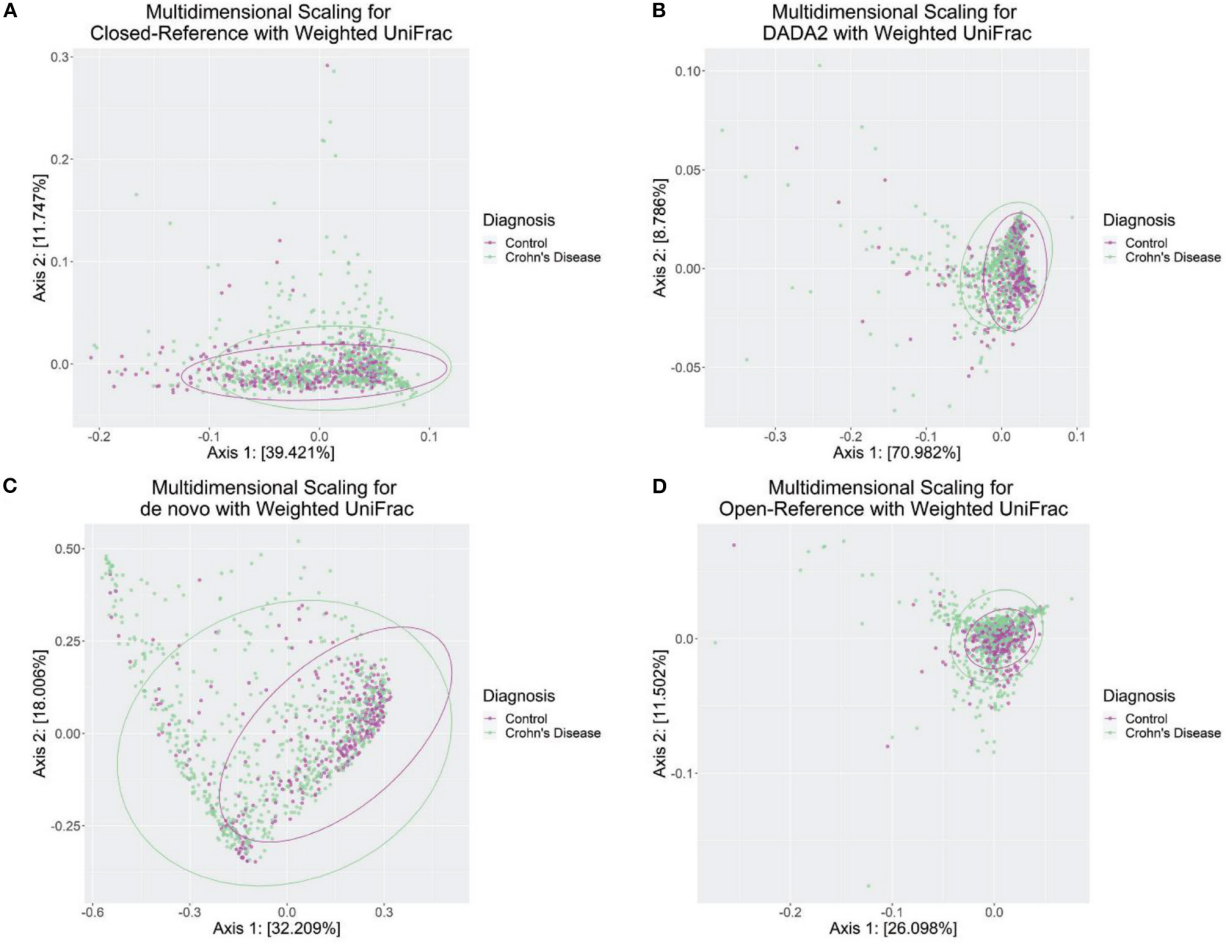
Weighted UniFrac ordination plot from four OTU/ASV assignment methods. **(A)** Closed-Reference, an OTU assignment method. **(B)** DADA2, an ASV assignment method. **(C)** *de novo*, an OTU assignment method. **(D)** Open-Reference, an OTU assignment method. The ellipses were drawn based on the multivariate *t*-distribution, respectively, for cases/controls.

We investigated how different OTU/ASV assignment methods react to the combination of a variety of ordination and the distance measure. The best performing OTU/ASV assignment method was DADA2, with the first and second axis separating 70.982 and 8.786% which means the combination of the first two axes explained roughly 80% of the total variance between Crohn's Disease and Control subjects (Figure 5B). While the other methods perform relatively well, DADA2 worked much better on distinguishing Crohn's Disease subjects with control with weighted UniFrac (Figures 5A,C,D).

Moreover, we followed through with the statistical tests to determine if the first two axes were significantly affected by the disease category between Crohn's Disease and Control. A previous study determined the usefulness of using the two axes from the Multidimensional scaling techniques to discriminate between the case and control cases, and with consistencies across different OTU assignment percentage matches, i.e., 99, 95, 90, and 85% (Frank et al., 2007). We followed a similar protocol and examined using different combinations of distance methods and OTU/ASV assignment methods; our results did not replicate the significant separation between Crohn's disease and control. However, we observe an adjusted *R*^2^ of 0.864 with a *p*-value of 0.081 using the Jaccard distance and *de novo* OTU assignment methods. The second-best test, PERMANOVA, uses weighted UniFrac on Closed-Reference OTU assignment methods with an adjusted *R*^2^ of 0.6525 and a *p*-value of 0.073. The full table is in Supplementary Table 4.

#### Relationship Between the Phylogenetic Trees Among Clustering Methods

**Figure 6:**
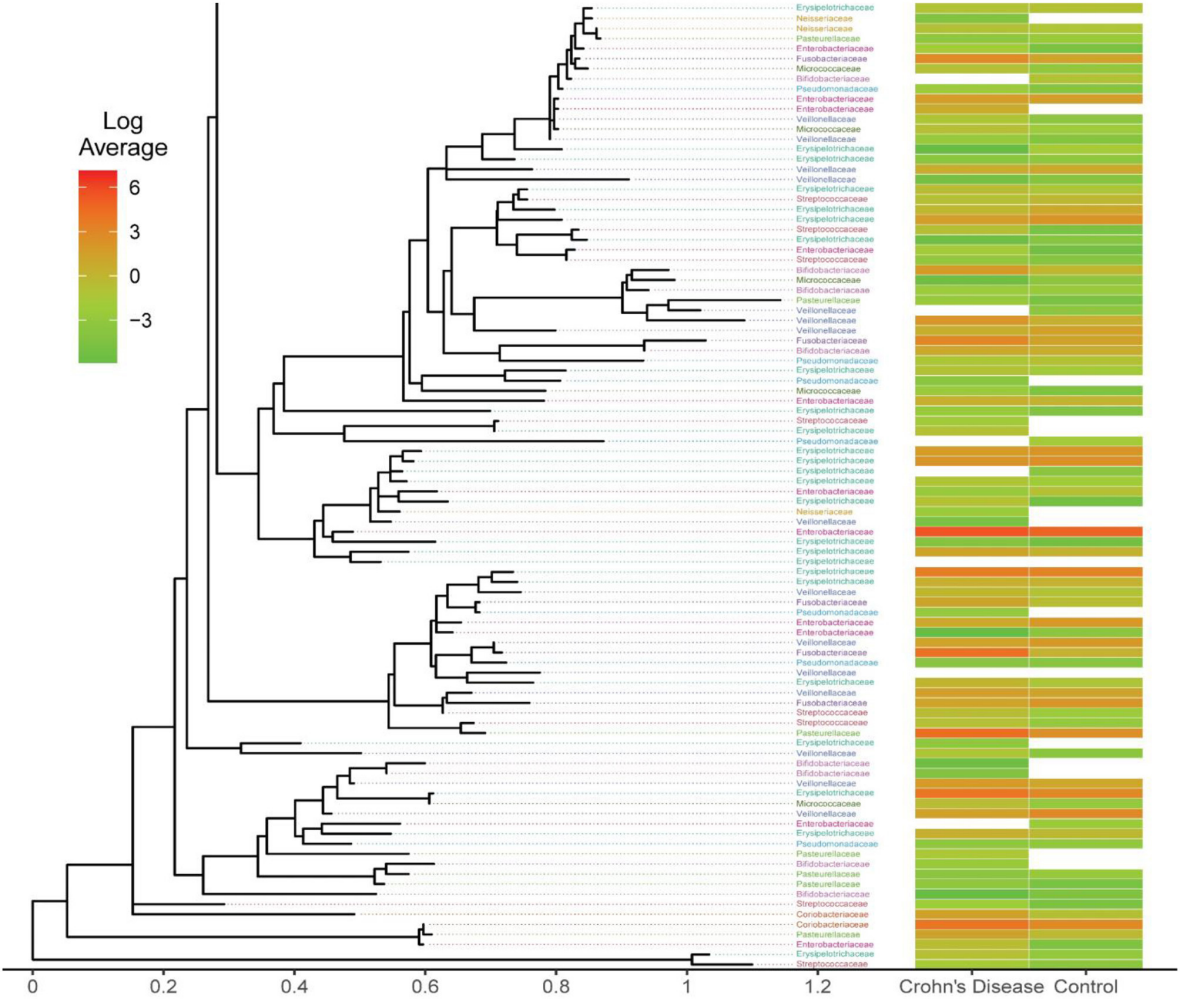
Phylogenetic tree from the open-reference clustering methods showing the mean log-transformed average count between the Crohn's Disease and Control. This is a subset of the full plot due to the large dimension of the original files. The full figure for all four OTU/ASV assignment methods are included in Supplementary Figure 9.

As we discussed earlier, the number of unique features reported by different OTU/ASV methods are different, so the resultant phylogenetic trees also differ. Here, we focus our investigation on the Family-level taxa, and we extracted the taxa from the eleven important taxa that previous studies had identified. We calculated the log-transformed average of Crohn's disease and Control OTU/ASV counts per the taxon assignment. Examining the taxonomic tree closely, we detected some unique taxon assignments from Crohn's disease group or control group. There are different observable patterns between the case and control (Figure 6), including the log-scale differences and the present/absent differences.

### k-mers

Finally, we examined two separate types of k-mers, the very short-chain k-mers (4, 5, 6, and 7-mers) and the short-chain k-mers (15, 21, 30-mers). Both very short-chain k-mers and short-chain k-mers, when combined with effective machine learning methods, perform as well as the top-ranked OTU/ASV clustering methods for host trait prediction. From the computation side, very short-chain k-mers can be calculated quickly by parsing the raw FASTQ files, but short-chain k-mers take longer to extract, and due to the enormous number of possible combinations, we filtered the count matrices to make the final table computationally feasible. Here, any unique-k-mers with fewer than 5 reads were removed. The advantage of short-chain k-mers is the potential of mapping back to genomic data to better understand the underlying biology (Koslicki and Falush, 2016). With the short-chain k-mers, we could study them by mapping them back to a 16S rRNA database and extract their taxonomic information. Using these mappings will be an interesting area to explore for future projects. Very-short k-mers cannot be mapped uniquely back to a reference database, as they are ubiquitous in all samples.

### Benchmarking

While computational cost is not the primary goal of this journal, we nevertheless conducted benchmarking investigation by using our best phenotype, Crohn's Disease. We evaluated the difference in terms of time consumption by running the 100 iterations of 5-fold validation for the eleven machine learning methods we tested on a single core. Overall, the results suggested Elastic Net and xgBoost are the most time-consuming (Supplementary Figure 10). Also, normalization did not cause significant computation changes, and filtered combinations generally cause slightly shorter computation time (Supplementary Figure 11).

### Variable Importance

For each of the machine learning method, we calculated the mean and standard deviation across all 500 rounds to evaluate importance of features, as defined in 2.4.1. These outputs are included in the Supplementary Tables 5–16 for Order, Family, and Genus level feature outputs, for each of the combinations, the mean and standard deviation for the features from the machine learning models are shown.

## Discussion

This article aims to explore and compare the different upstreaming process and how they can affect downstream machine learning predictions. Despite the introduction of a large number of data pre-processing steps and machine learning methods, there has been little systematic exploration of the massive number of possible combinations of these approaches. While many of our findings accord with earlier smaller explorations, the definitive nature of our combination “search-space” provides important assurance that the community is applying generally best-practice methods for host-trait prediction. All of the completed combinations can be explored in the Shiny application in terms of their corresponding AUROC curve.

Firstly, we reviewed the impact of filtering and normalization on four OTU/ASV assignment methods. Normalization has only a modest impact on the downstream machine learning algorithm performance, while filtering has a more impact on overall performance of the algorithms. We also observed that the filtering criteria might throw out important taxa that had been identified as important from discovery studies. Depending on the goal of the machine learning methods, filtering criteria might need to be adjusted.

We also explored the usefulness of short-chain and very short-chain k-mers and their ability to differentiate between diseases and controls. Both types of k-mers can provide high-quality predictions that are equally as good as Genus and OTU/ASV assignment methods. This area needs further research to uncover additional potential of using k-mers as predictors.

While we tried many combinations of different processing steps, it is impossible to consider all scenarios, and there are limitations to our conclusions and in the available data. Both of the datasets we used are based on 16S rRNA from the V4 hypervariable region. Previous studies have shown that other hypervariable regions, or a combination of variable regions, affects biodiversity and community state types, which could eventually cause differences in prediction accuracy (Graspeuntner et al., 2018; Bukin et al., 2019). Moreover, the choice of the reference database may also affect the quality of the OTU/ASV assignment results, and it is recommended to use a curated database. Lastly, we employed only a single combination of filtering criteria, and different studies might require more exclusive or inclusive filtering standards, depending on the disease of interest. The current filtering criteria focus on removing rare taxonomic features.

Overall, we provided a comprehensive comparison of commonly used machine learning algorithms and how upstream methods affect overall outcomes.

## Data Availability Statement

Publicly available datasets were analyzed in this study. This data can be found here: The raw data for the 16s rRNA datasets were downloaded from European Nucleotide Archive - IBD Dataset (PRJEB13679), TwinsUK Dataset (PRJEB6702 and PRJEB6705). The BMI data for the TwinsUK dataset is from the Goodrich dataset (Goodrich et al., 2014).

## Author Contributions

Y-HZ is the leader of this study and contributed to writing the manuscript, designing the data analysis, summarizing the result, and software management. KS contributed to writing the manuscript, data processing, data analysis, and results summary. All authors contributed to the article and approved the submitted version.

## Conflict of Interest

The authors declare that the research was conducted in the absence of any commercial or financial relationships that could be construed as a potential conflict of interest.
