# Supplementary material for: Systematic Comparisons for Composition Profiles, Taxonomic Levels, and Machine Learning Methods for Microbiome-Based Disease Prediction
Source: Front Mol Biosci. 2020 Dec 16;7:610845. doi: 10.3389/fmolb.2020.610845 (PMC7772236; doi:10.3389/fmolb.2020.610845)
Supplement: Supplementary file 1 [file Presentation_1.pdf]

Supplementary Material:

Systematic Comparisons for Composition Profiles, Taxonomic Levels, and Machine Learning Methods for Microbiome-based Disease Prediction

Kuncheng Song[1], Fred A. Wright[2], Yi-Hui Zhou[3,*]

[1]Bioinformatics Research Center, North Carolina State University, Raleigh, NC, USA

[2]Departments of Statistics and Biological Sciences, North Carolina State University, Raleigh, NC, USA

[3]Department of Biological Sciences, North Carolina State University, Raleigh, NC, USA

*** Correspondence:**
Yi-Hui Zhou

yihui_zhou@ncsu.edu

1 DATA PROCESSING

1.1 Stage 1 – OTUs, ASVs, and K-mers Generation

The raw sequences first undergo FastQC to check the read qualities; there were no bad reads with quality scores below 20. Thus, all reads were subsequently imported to QIIME2. These demultiplexed reads undergo chimeric sequences removal as part of the QIIME2 (Version 2019.10) 16S rRNA analysis pipeline (Bolyen et al., 2019). The *de novo*, open-reference, and closed-reference were performed all with a 97% similarity using QIIME2 version 2019.10 (Bolyen et al., 2019). Similarly, DADA2 was also performed using QIIME2 using the demultiplexed reads directly (Bolyen et al., 2019; Callahan et al., 2016). All these four methods used the same reference provided by the SILVA rRNA database project Version 132 release (Quast et al., 2013). The final output from these procedures is four OTUs/ASVs-level tables, and we subsequently extract the higher level OTUs/ASVs count matrices. We have also extracted seven levels of OTUs/ASVs matrices based on their taxonomic assignments: phylum, class, order, family, genus, species, and OTUs/ASV. We also removed three taxonomy assignments from these count matrices: missing, ambiguous taxa, or unassigned. The full processing workflow is shown in Figure **S1**.

Raw k-mer calculations were done using R (Version 4.0.1) (R Core Team, 2013) by breakdown each of the FASTQ raw sequences and counts each of the fragments to a raw counting matrix. We split the k-mers length in our example to very short k-mers and short k-mers, the very short k-mers are k-mers range from 4-mers, 5-mers, 6-mers, and 7-mers, and the short k-mers are 15-mers, 21-mers, and 30mers. Both k-mer categories have been used to study for their phenotype prediction properties (Asgari et al., 2018; Molik et al., 2020). Since we are trying to preserve as many unique reads and

their associated counts as possible, and at the same time, make the analyses computationally feasible, during the k-mer extraction process for the short k-mers, we have removed any reads less than five counts out of each sample. This process reduces the resulted dataset and dimension by at least half.

Through this stage, we have generated 21 OTUs count matrices, 7 ASV count matrices, 4 very short-chain k-mer matrices, and 3 short-chain k-mer matrices.

1.2 Stage 2 Normalization

Previous publications have investigated the potential usefulness of utilizing normalization technology on the count matrices (Weiss et al., 2017). This idea stemmed from the normalization of RNA-Seq data, which is another large count matrix similar to OTUs/ASVs matrices. The key difference is the OTUs table is zero-inflated, which is not a common problem in the RNA-Seq data (except single-cell RNA-Seq). Thus, some of the methods used for RNA-Seq transformation might benefit from adjustment, which is an active research field. In this project, we choose DESeq2, which was recommended from a previous publication, though its usefulness from simulated data was controversial (McMurdie & Holmes, 2014; Weiss et al., 2017). Briefly, DESeq2 models the counts with the Negative Binomial to detect the differential abundance while accounting for the sampling depth and OTUs/ASVs composition. Due to the large dimension of the short-chain k-mers, which are the 15-mer, 21-mer, and 30-mer, and we removed the count lower than 5 reads per sample when generating the final count matrices.

After this stage, we have obtained 42 OTUs count matrices, 14 ASV count matrix, 8 very short-chain k-mer matrices, and 3 short-chain k-mer matrices.

1.3 Stage 3 Filtering the Samples

For the OTUs and ASVs, count matrices undergo the three filtering criteria reported previously (Duvallet et al., 2017; Goodrich et al., 2014; Zhou & Gallins, 2019). The first filter excludes the sample with less than 100 reads, and the second filter subtracts OTUs with less than 10 reads. (Duvallet et al., 2017) The third filter removes OTUs that present less than 5% of samples (Goodrich et al., 2014). We detail the number of features prior to the machine learning algorithm is shown in table **Supplementary Table 1**. Generally, the filtering removed fewer features (OTUs/ASVs) with a more specific taxonomic level, i.e., more features were kept on the species level compared to class level. There are many ways that filtering can undergo; other studies have implemented a less rigorous third rule, which only removed OTUs that present less than 1% of samples (Ross et al., 2015; Singh et al., 2015; Vincent et al., 2013).

After this stage, we have obtained 84 OTUs count matrices, 28 ASV count matrix, 8 very short-chain k-mer matrices, and 3 short-chain k-mer matrices.

1.4 Stage 4 Machine Learning Methods

The details for most of the machine learning methods, except logistic regression, were part of our previous work and explained in great detail previously (Zhou & Gallins, 2019). All the methods were tested against a binary outcome, Disease Vs. Control.

Among the 11 machine learning algorithms we have tested, ten methods were part of the supervised learning methods. The LASSO (Tibshirani, 1996), Ridge (Hoerl & Kennard, 1970), Elastic Net (Zou

& Hastie, 2005), and Logistic (Nelder., 1989) are the regression aspects of the supervised learning. The Support Vector Machine (SVM)(Cortes & Vapnik, 1995), Gradient Boost (xgBoost) (Friedman, 1999), random forest (Breiman, 2001), K-nearest Neighbors, Hierarchical clustering, and Neural Network (Ditzler et al., 2015) represents different methods within the supervised classification category. Lastly, we also used a K-means method to shed some light on unsupervised machine learning methods.

After this stage, our analyses have undergone 924 OTUs-based predictions, 308 ASV-based predictions, 88 very short-chain k-mer-based predictions, and 33 short-chain k-mer-based predictions. This sums up to a total of 1,353 combinations per disease type.

1.5 Stage 5 Evaluation

K-Fold cross-validation is a commonly used resampling protocol for evaluating machine learning methods. In our pipeline, we utilize a 5-fold cross-validation scheme with 100 iterations. Briefly, at the beginning of each iteration, we randomly break down the sample consists of the diseased and control subjects into five roughly equal-size groups. Then each of these five groups was used as the testing set, where the remaining four groups were selected as the training set. At the end of each 5-fold validation, we extract all the predicted values from each of the 11 methods and save them prior to running the next iterations. For the TwinsUK dataset, we modified the sampling methods to ensure twins who came from the same family are kept in the same training/testing set.

Overall, we have just over 5,412 combinations tested.

Reference:

Asgari, E., Garakani, K., McHardy, A. C., & Mofrad, M. R. K. (2018). MicroPheno: predicting environments and host phenotypes from 16S rRNA gene sequencing using a k-mer based representation of shallow sub-samples. *Bioinformatics (Oxford, England)*, *34*(13), i32–i42. https://doi.org/10.1093/bioinformatics/bty296

Bolyen, E., Rideout, J. R., Dillon, M. R., Bokulich, N. A., Abnet, C. C., Al-Ghalith, G. A., Alexander, H., Alm, E. J., Arumugam, M., Asnicar, F., Bai, Y., Bisanz, J. E., Bittinger, K., Brejnrod, A., Brislawn, C. J., Brown, C. T., Callahan, B. J., Caraballo-Rodríguez, A. M., Chase, J., … Caporaso, J. G. (2019). Reproducible, interactive, scalable and extensible microbiome data science using QIIME 2. *Nature Biotechnology*, *37*(8), 852–857. https://doi.org/10.1038/s41587-019-0209-9

Breiman, L. (2001). *Random Forests* (Vol. 45).

Callahan, B. J., McMurdie, P. J., Rosen, M. J., Han, A. W., Johnson, A. J. A., & Holmes, S. P. (2016). DADA2: High-resolution sample inference from Illumina amplicon data. *Nature Methods*, *13*(7), 581–583. https://doi.org/10.1038/nmeth.3869

Cortes, C., & Vapnik, V. (1995). Support-vector networks. *Machine Learning*, *20*(3), 273–297. https://doi.org/10.1007/bf00994018

Ditzler, G., Polikar, R., & Rosen, G. (2015). Multi-Layer and Recursive Neural Networks for Metagenomic Classification. *IEEE Transactions on Nanobioscience*, *14*(6), 608–616. https://doi.org/10.1109/TNB.2015.2461219

Duvallet, C., Gibbons, S. M., Gurry, T., Irizarry, R. A., & Alm, E. J. (2017). Meta-analysis of gut microbiome studies identifies disease-specific and shared responses. *Nature Communications*, *8*(1). https://doi.org/10.1038/s41467-017-01973-8

Friedman, J. H. (1999). Greedy Function Approximation : A Gradient Boosting Machine 1 Function estimation 2 Numerical optimization in function space. *North*, *1*(3), 1–10. https://doi.org/10.2307/2699986

Goodrich, J. K., Waters, J. L., Poole, A. C., Sutter, J. L., Koren, O., Blekhman, R., Beaumont, M., Treuren, W. Van, Knight, R., Bell, J. T., Spector, T. D., Clark, A. G., & Ley, R. E. (2014). Human genetics shape the gut microbiome. *Cell*, *159*(4), 789. https://doi.org/10.1016/J.CELL.2014.09.053

Hoerl, A. E., & Kennard, R. W. (1970). Ridge Regression: Applications to Nonorthogonal Problems. *Technometrics*, *12*(1), 69–82. https://doi.org/10.1080/00401706.1970.10488635

McMurdie, P. J., & Holmes, S. (2014). Waste Not, Want Not: Why Rarefying Microbiome Data Is Inadmissible. *PLoS Computational Biology*, *10*(4), e1003531. https://doi.org/10.1371/journal.pcbi.1003531

Molik, D. C., Pfrender, M. E., & Emrich, S. J. (2020). Uncovering Effects from the Structure of Metabarcode Sequences for Metagenetic and Microbiome Analysis. *Methods and Protocols*, *3*(1), 22. https://doi.org/10.3390/mps3010022

Nelder., P. M. and J. A. (1989). *Generalized linear models*. London ; New York : Chapman and Hall, 1989. https://catalog.lib.ncsu.edu/catalog/NCSU4818332

Quast, C., Pruesse, E., Yilmaz, P., Gerken, J., Schweer, T., Yarza, P., Peplies, J., & Glöckner, F. O. (2013). The SILVA ribosomal RNA gene database project: improved data processing and web-based tools. *Nucleic Acids Research*, *41*(Database issue), D590-6. https://doi.org/10.1093/nar/gks1219

R Core Team. (2013). *R: A language and environment for statistical computing*. http://cran.univ-paris1.fr/web/packages/dplR/vignettes/intro-dplR.pdf

Ross, M. C., Muzny, D. M., McCormick, J. B., Gibbs, R. A., Fisher-Hoch, S. P., & Petrosino, J. F. (2015). 16S gut community of the Cameron County Hispanic Cohort. *Microbiome*, *3*(1). https://doi.org/10.1186/s40168-015-0072-y

Singh, P., Teal, T. K., Marsh, T. L., Tiedje, J. M., Mosci, R., Jernigan, K., Zell, A., Newton, D. W., Salimnia, H., Lephart, P., Sundin, D., Khalife, W., Britton, R. A., Rudrik, J. T., & Manning, S. D. (2015). Intestinal microbial communities associated with acute enteric infections and disease recovery. *Microbiome*, *3*(1), 45. https://doi.org/10.1186/s40168-015-0109-2

Tibshirani, R. (1996). Regression Shrinkage and Selection Via the Lasso. *Journal of the Royal Statistical Society: Series B (Methodological)*, *58*(1), 267–288. https://doi.org/10.1111/j.2517-6161.1996.tb02080.x

Vincent, C., Stephens, D. A., Loo, V. G., Edens, T. J., Behr, M. A., Dewar, K., & Manges, A. R.

(2013). Reductions in intestinal Clostridiales precede the development of nosocomial Clostridium difficile infection. *Microbiome*, *1*(1), 18. https://doi.org/10.1186/2049-2618-1-18

Weiss, S., Xu, Z. Z., Peddada, S., Amir, A., Bittinger, K., Gonzalez, A., Lozupone, C., Zaneveld, J. R., Vázquez-Baeza, Y., Birmingham, A., Hyde, E. R., & Knight, R. (2017). Normalization and microbial differential abundance strategies depend upon data characteristics. *Microbiome*, *5*(1), 27. https://doi.org/10.1186/s40168-017-0237-y

Zhou, Y.-H., & Gallins, P. (2019). A Review and Tutorial of Machine Learning Methods for Microbiome Host Trait Prediction. *Frontiers in Genetics*, *10*(JUN), 579. https://doi.org/10.3389/fgene.2019.00579

Zou, H., & Hastie, T. (2005). Regularization and variable selection via the elastic net. *Journal of the Royal Statistical Society: Series B (Statistical Methodology)*, *67*(2), 301–320. https://doi.org/10.1111/J.1467-9868.2005.00503.X@10.1111/(ISSN)1467-9868.TOP_SERIES_B_RESEARCH

2 SUPPLEMENTARY FIGURES

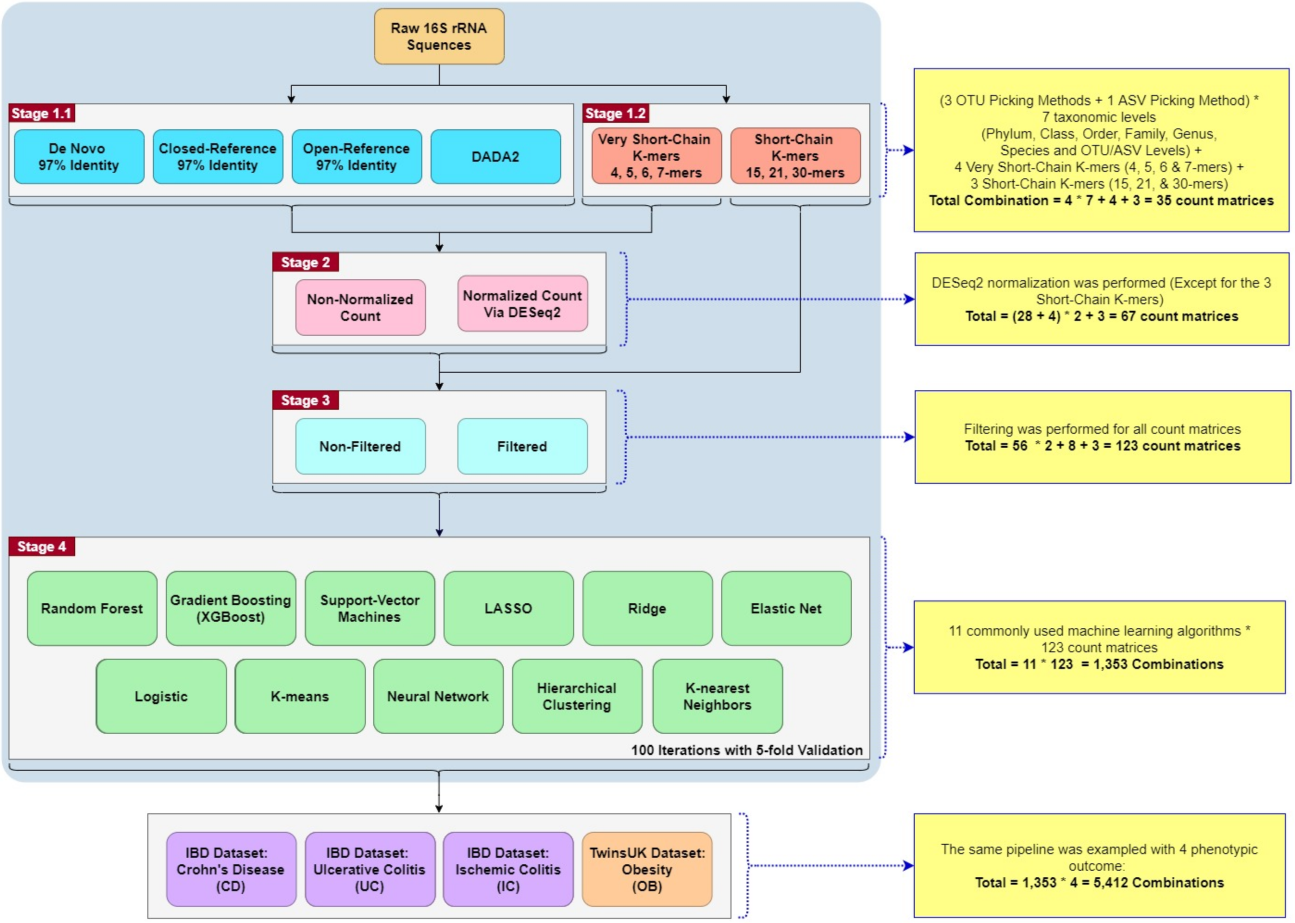

Figure S1. The workflow of the projects with the detailed calculation of count matrices or combinations at each stage.

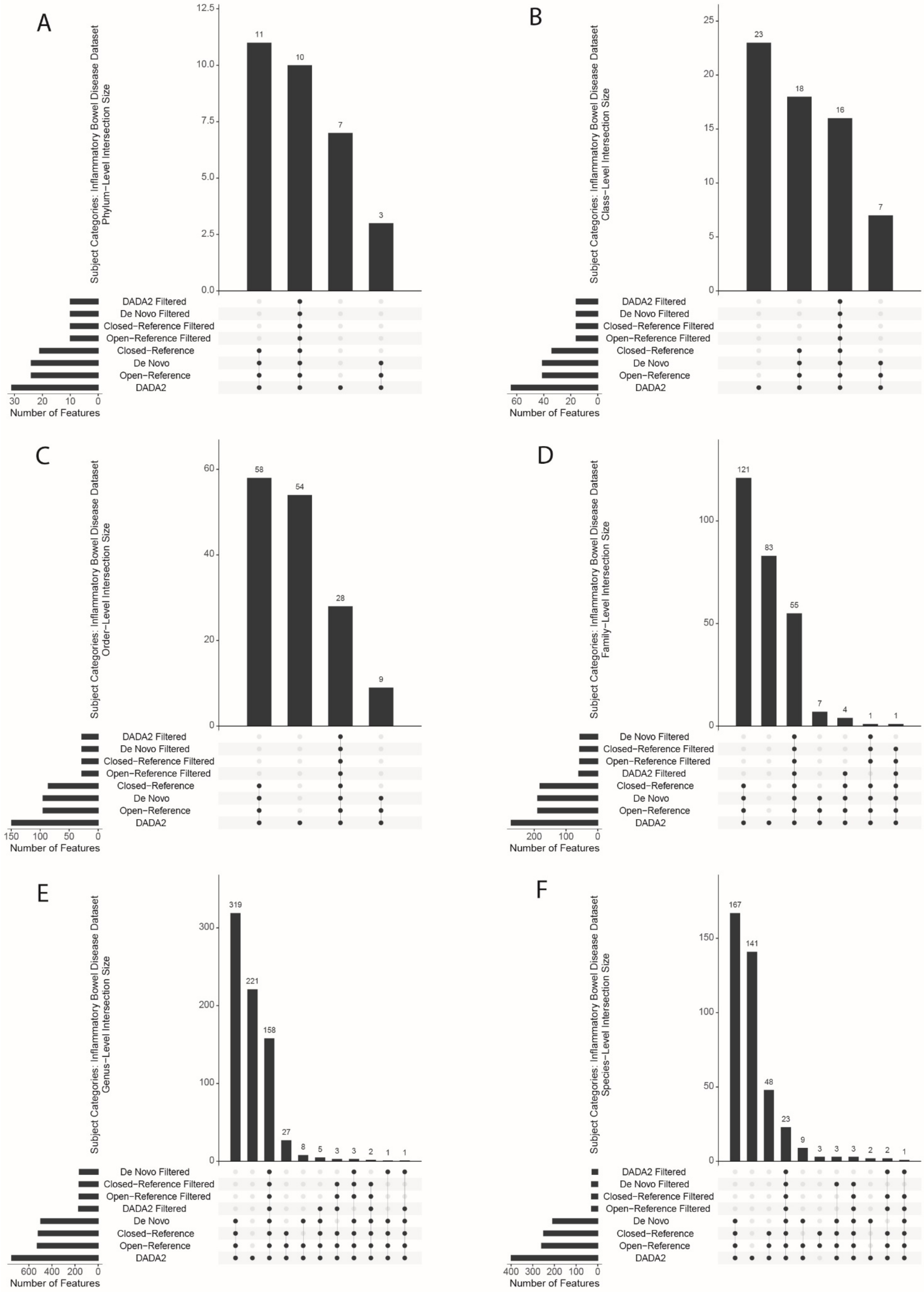
A
Subject Categories: Inflammatory Bowel Disease Dataset
Phylum−Level Intersection Size
11
10
7
3
B
Subject Categories: Inflammatory Bowel Disease Dataset
Class−Level Intersection Size
23
18
16
7
C
Subject Categories: Inflammatory Bowel Disease Dataset
Order−Level Intersection Size
58
54
28
9
D
Subject Categories: Inflammatory Bowel Disease Dataset
Family−Level Intersection Size
121
83
55
7
4
1
1
E
Subject Categories: Inflammatory Bowel Disease Dataset
Genus−Level Intersection Size
319
221
158
27
8
5
3
3
2
1
1
F
Subject Categories: Inflammatory Bowel Disease Dataset
Species−Level Intersection Size
167
141
48
23
9
3
3
3
2
2
1
DADA2 Filtered
De Novo Filtered
Closed−Reference Filtered
Open−Reference Filtered
Closed−Reference
De Novo
Open−Reference
DADA2
Number of Features

Figure S2. Upset plot for the interaction of features for all three Inflammatory Bowel Disease diagnoses and Control. Including the filtered and non-filtered OTU/ASV picking methods at different taxonomic levels. **(A-F)** Phylum, Class, Order, Family, Genus, and Species-level.

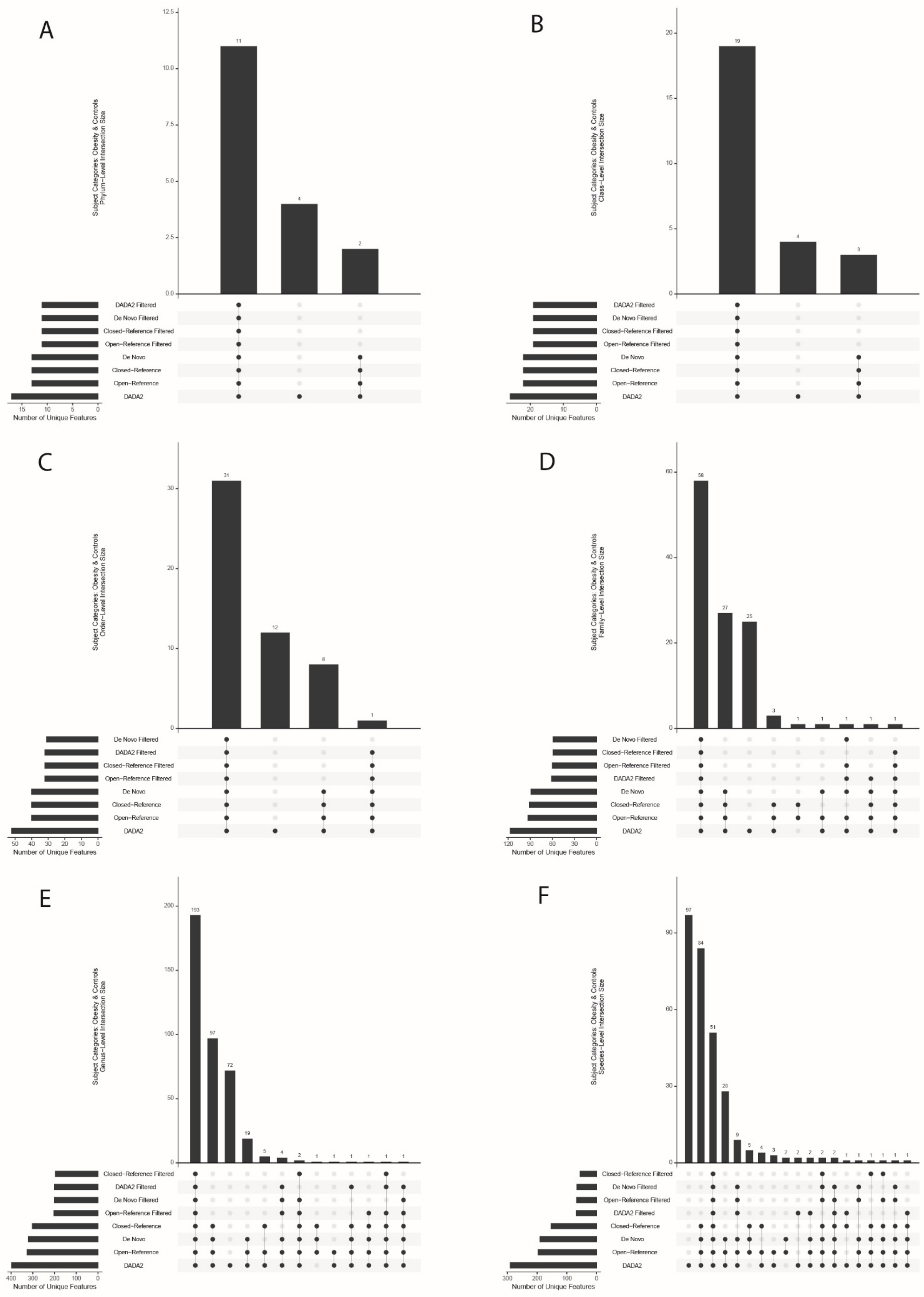
A
Subject Categories: Obesity & Controls
Phylum−Level Intersection Size
DADA2 Filtered
De Novo Filtered
Closed−Reference Filtered
Open−Reference Filtered
De Novo
Closed−Reference
Open−Reference
DADA2
Number of Unique Features
B
Subject Categories: Obesity & Controls
Class−Level Intersection Size
C
Subject Categories: Obesity & Controls
Order−Level Intersection Size
D
Subject Categories: Obesity & Controls
Family−Level Intersection Size
E
Subject Categories: Obesity & Controls
Genus−Level Intersection Size
F
Subject Categories: Obesity & Controls
Species−Level Intersection Size

Figure S3. Upset plot for the interaction of features for Obesity and Control. Including the filtered and non-filtered OTU/ASV picking methods at different taxonomic levels. **(A-F)** Phylum, Class, Order, Family, Genus, and Species-level.

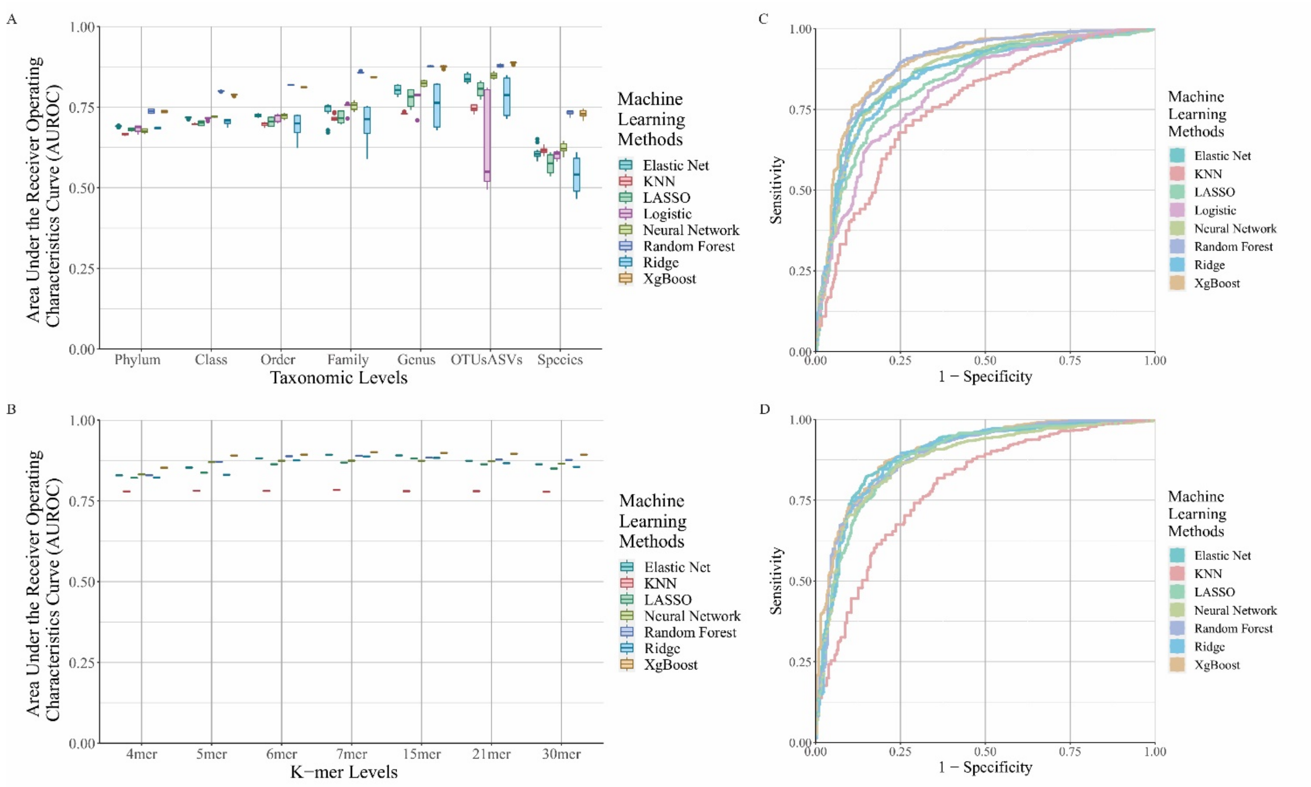

Figure S4. The area under the ROC curve (AUROC) for selected machine learning methods across different taxonomic levels and k-mer lengths for Crohn's Disease. A. Boxplots of the AUROC for eight machine learning methods from OTU/ASV assignment methods across all seven taxonomic levels. **B**. Boxplots of the AUROC for seven machine learning methods across different k-mer lengths. The consistently top-performing methods random forest and xgBoost are highlighted in green and orange, respectively. **C**. Top-performing combinations and their corresponding ROC for each of the eight machine learning methods from OTU/ASV assignment methods across all seven taxonomic levels. Hierarchical clustering, K-means, and Support Vector Machine were removed from the figure due to their poor performance. **D** Top-performing combinations and their corresponding ROC for the seven machine learning methods across different k-mer lengths. For the OTU/ASV assignment methods, hierarchical clustering, k-means, and support vector machine are removed from the figure due to their poor performance. For the k-mer based methods, hierarchical clustering, k-means, support vector machine, and logistic regression were removed from the figure due to their poor performance.

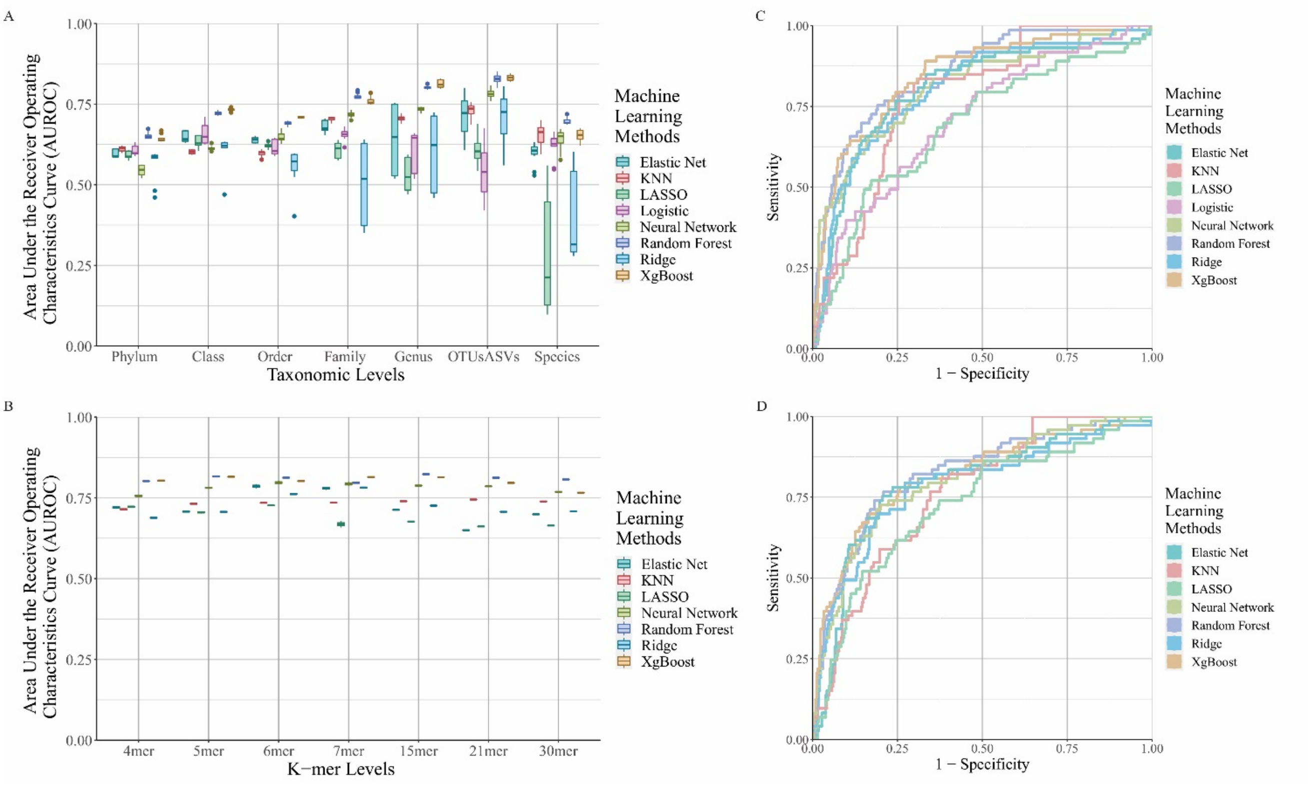

Figure S5. The area under the ROC curve (AUROC) for selected machine learning methods across different taxonomic levels and k-mer lengths for Interstitial Cystitis. A. Boxplots of the AUROC for eight machine learning methods from OTU/ASV assignment methods across all seven taxonomic levels. **B**. Boxplots of the AUROC for seven machine learning methods across different k-mer lengths. The consistently top-performing methods random forest and xgBoost are highlighted in green and orange, respectively. **C**. Top-performing combinations and their corresponding ROC for each of the eight machine learning methods from OTU/ASV assignment methods across all seven taxonomic levels. Hierarchical clustering, K-means, and Support Vector Machine were removed from the figure due to their poor performance. **D** Top-performing combinations and their corresponding ROC for the seven machine learning methods across different k-mer lengths. For the OTU/ASV assignment methods, hierarchical clustering, k-means, and support vector machine are removed from the figure due to their poor performance. For the k-mer based methods, hierarchical clustering, k-means, support vector machine, and logistic regression were removed from the figure due to their poor performance.

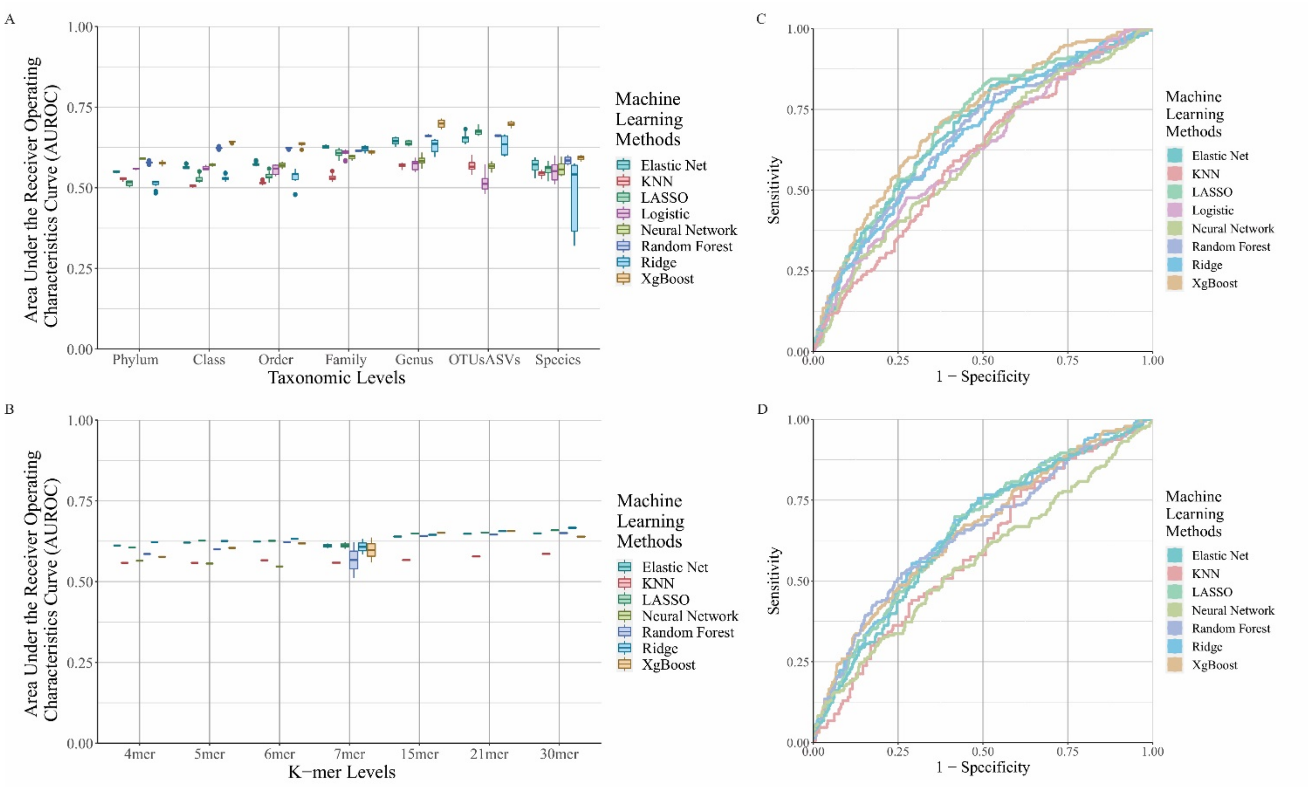

Figure S6. The area under the ROC curve (AUROC) for selected machine learning methods across different taxonomic levels and k-mer lengths for Obesity. A. Boxplots of the AUROC for eight machine learning methods from OTU/ASV assignment methods across all seven taxonomic levels. **B**. Boxplots of the AUROC for seven machine learning methods across different k-mer lengths. The consistently top-performing methods random forest and xgBoost are highlighted in green and orange, respectively. **C**. Top-performing combinations and their corresponding ROC for each of the eight machine learning methods from OTU/ASV assignment methods across all seven taxonomic levels. Hierarchical clustering, K-means, and Support Vector Machine were removed from the figure due to their poor performance. **D** Top-performing combinations and their corresponding ROC for the seven machine learning methods across different k-mer lengths. For the OTU/ASV assignment methods, hierarchical clustering, k-means, and support vector machine are removed from the figure due to their poor performance. For the k-mer based methods, hierarchical clustering, k-means, support vector machine, and logistic regression were removed from the figure due to their poor performance.

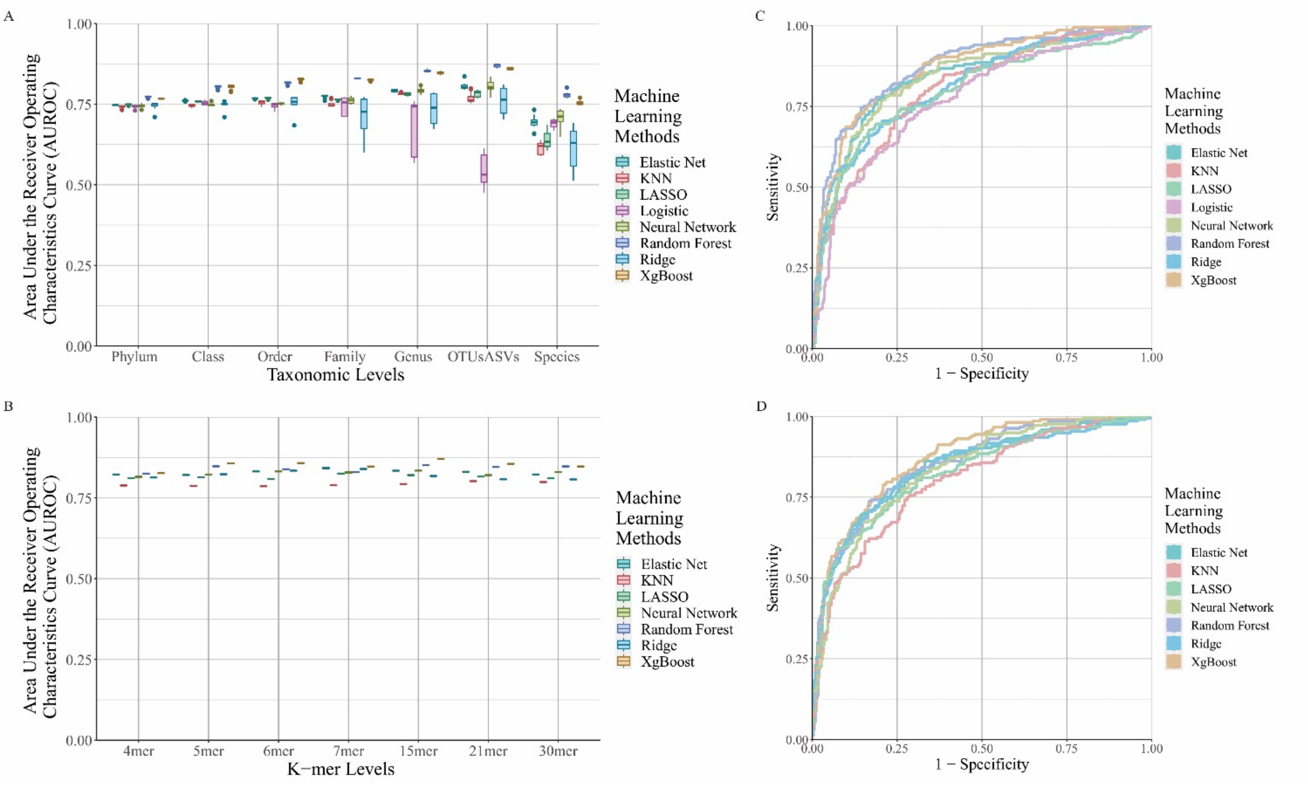

Figure S7. The area under the ROC curve (AUROC) for selected machine learning methods across different taxonomic levels and k-mer lengths for Ulcerative Colitis. A. Boxplots of the AUROC for eight machine learning methods from OTU/ASV assignment methods across all seven taxonomic levels. **B**. Boxplots of the AUROC for seven machine learning methods across different k-mer lengths. The consistently top-performing methods random forest and xgBoost are highlighted in green and orange, respectively. **C**. Top-performing combinations and their corresponding ROC for each of the eight machine learning methods from OTU/ASV assignment methods across all seven taxonomic levels. Hierarchical clustering, K-means, and Support Vector Machine were removed from the figure due to their poor performance. **D** Top-performing combinations and their corresponding ROC for the seven machine learning methods across different k-mer lengths. For the OTU/ASV assignment methods, hierarchical clustering, k-means, and support vector machine are removed from the figure due to their poor performance. For the k-mer based methods, hierarchical clustering, k-means, support vector machine, and logistic regression were removed from the figure due to their poor performance.

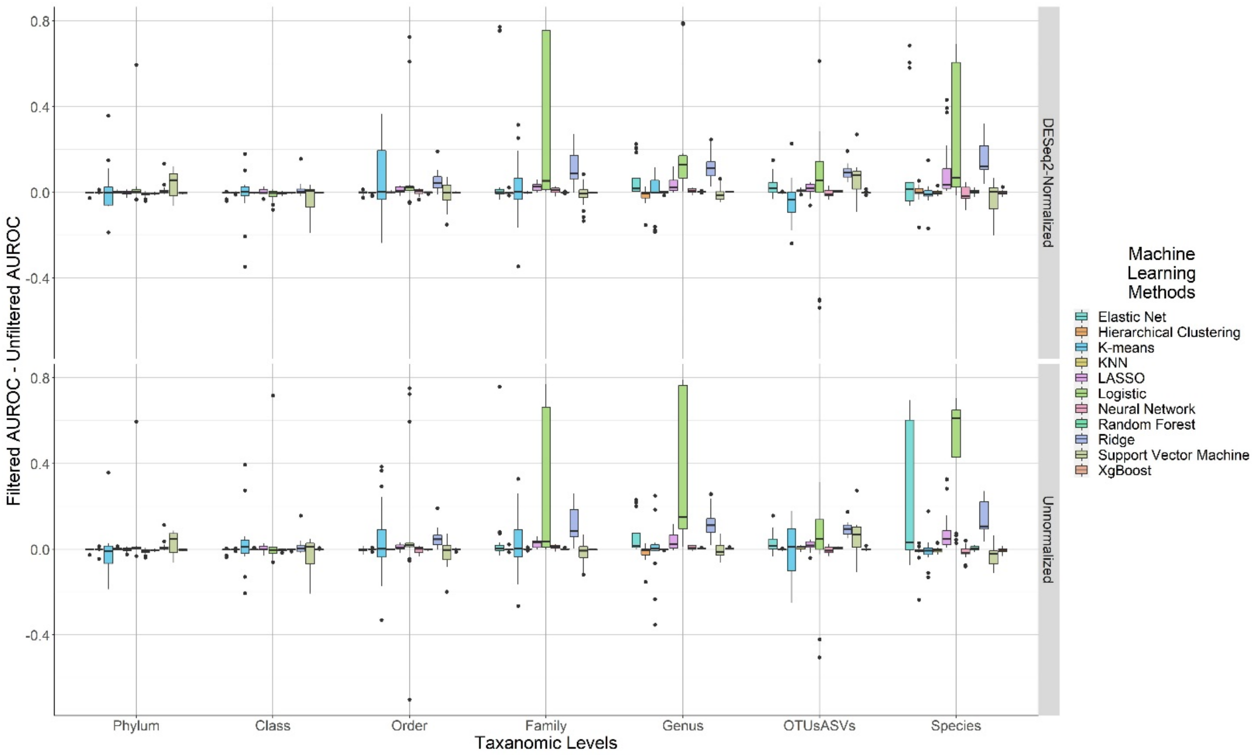

Figure S8. The Difference in AUROC Caused by Filtering between Normalized and Unnormalized Combinations. The Y-axis is the difference between the filtered and unfiltered AUROC when holding other parameters at the same level. If the results are above 0, it means the filtered combination generated a better AUROC compared to the unfiltered combination. The top row is the results from DESeq2 normalization. The bottom row is the result of unnormalized combinations.

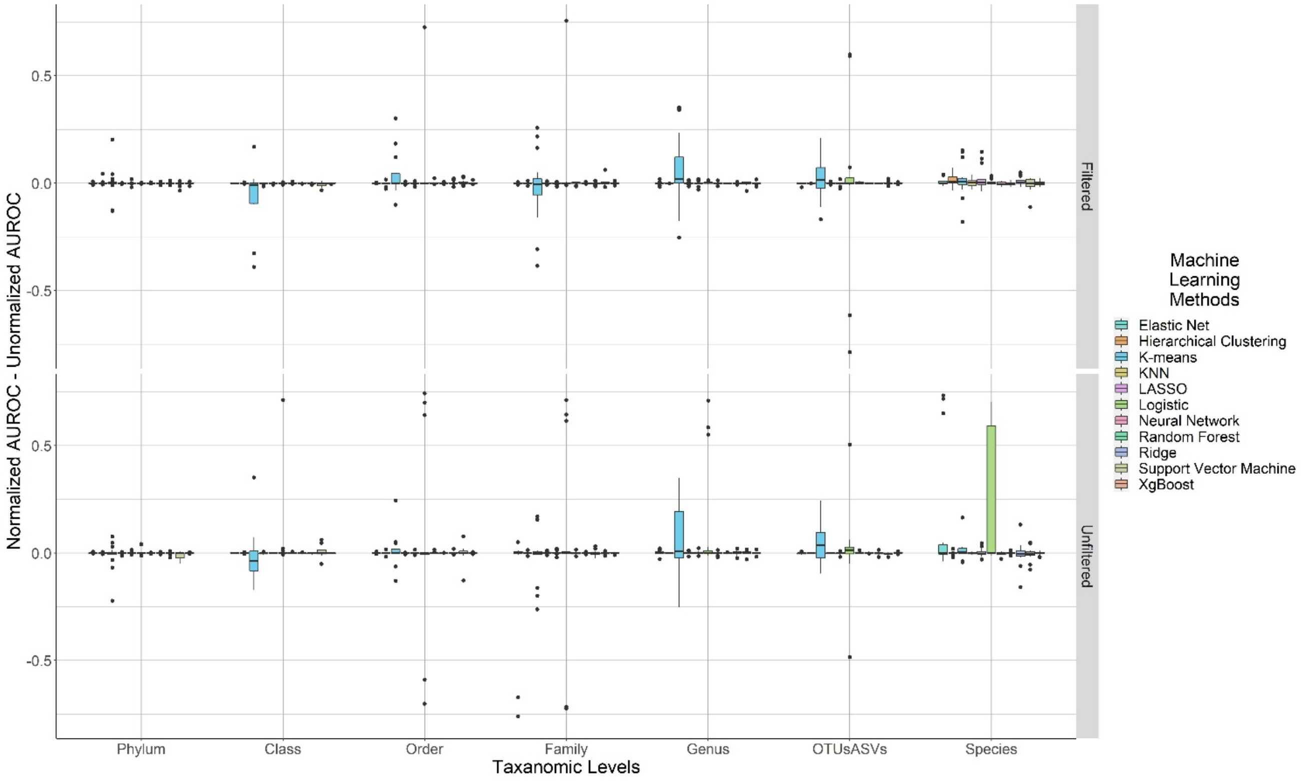

Figure S9. The Difference in AUROC Caused by Normalization between Filtered and Unfiltered Combinations. The Y-axis is the difference between the normalized and unnormalized AUROC when holding other parameters at the same level. If the results are above 0, it means the normalized combination generated a better AUROC compared to the unnormalized combination. The top row is the results from filtered combinations. The bottom row is the result of unfiltered combinations.

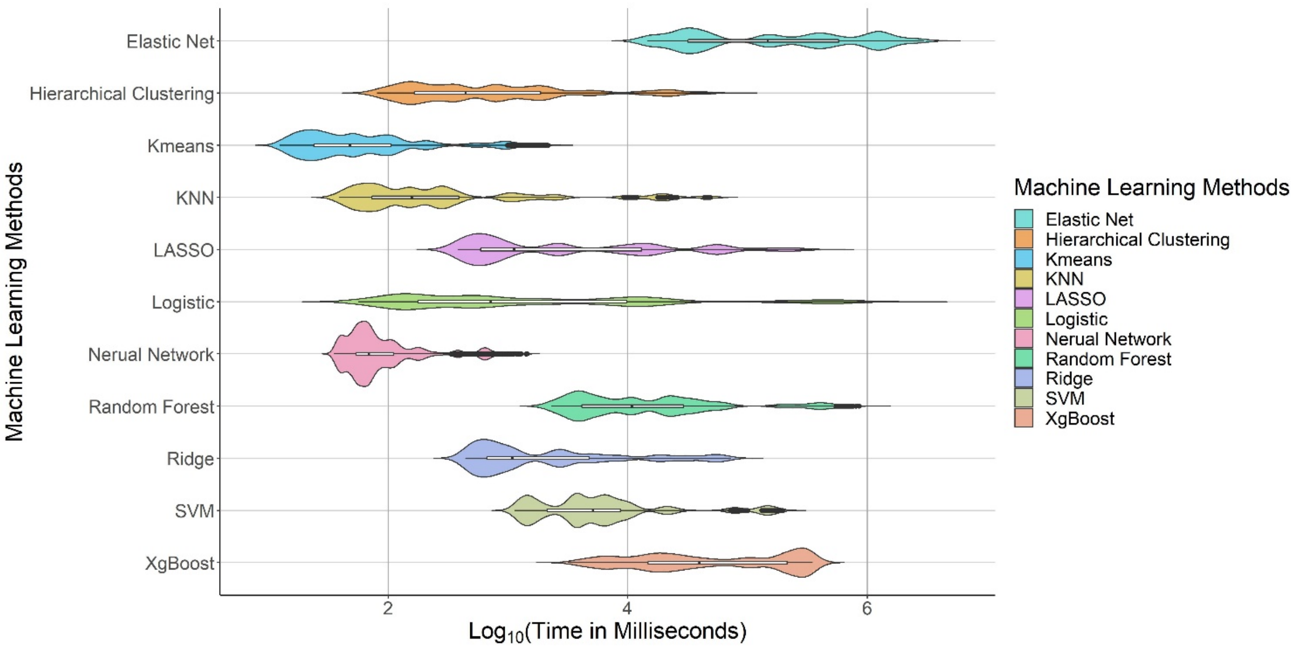

Figure S10. Benchmarking the Machine Learning Algorithms. The X-axis is the $\log_{10}$-transformed milliseconds.

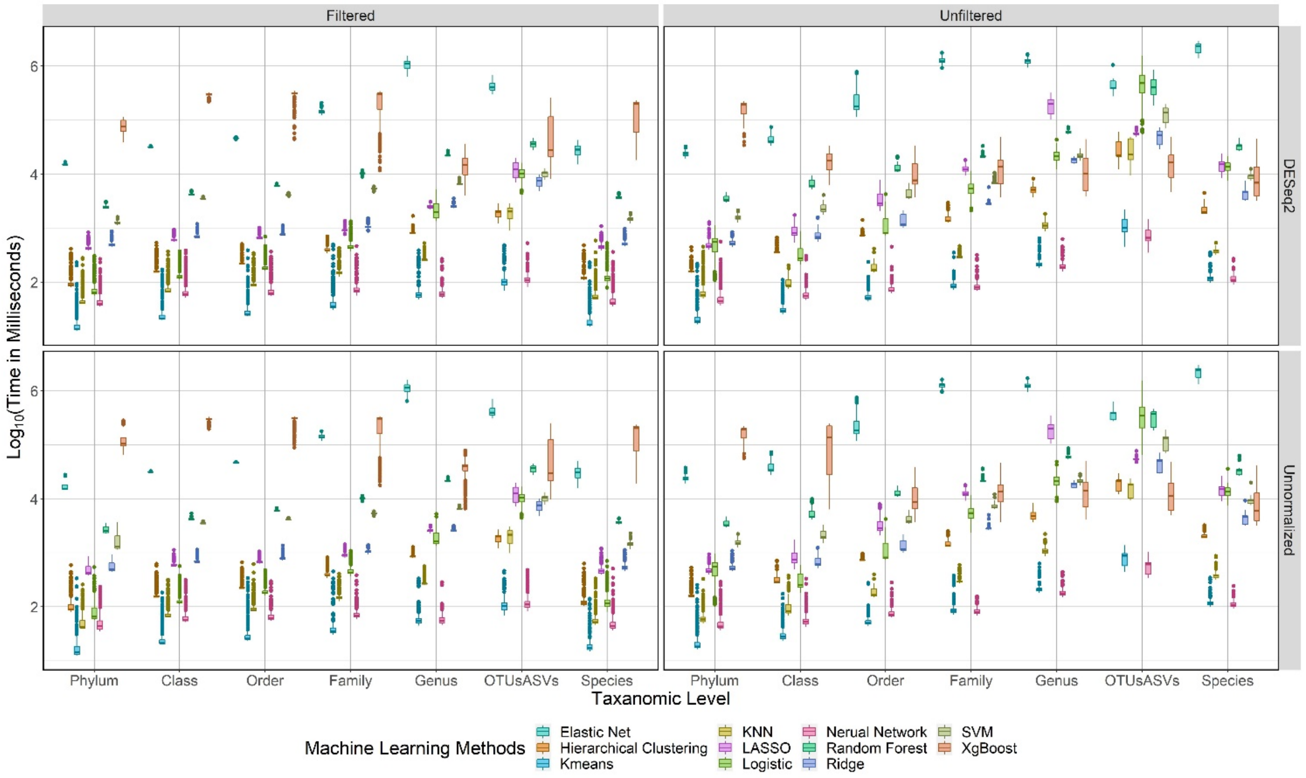

Figure S11. Benchmarking of machine learning algorithms faceted by the normalization and filtering status. TheY-axis is the $\log_{10}$-transformed milliseconds.

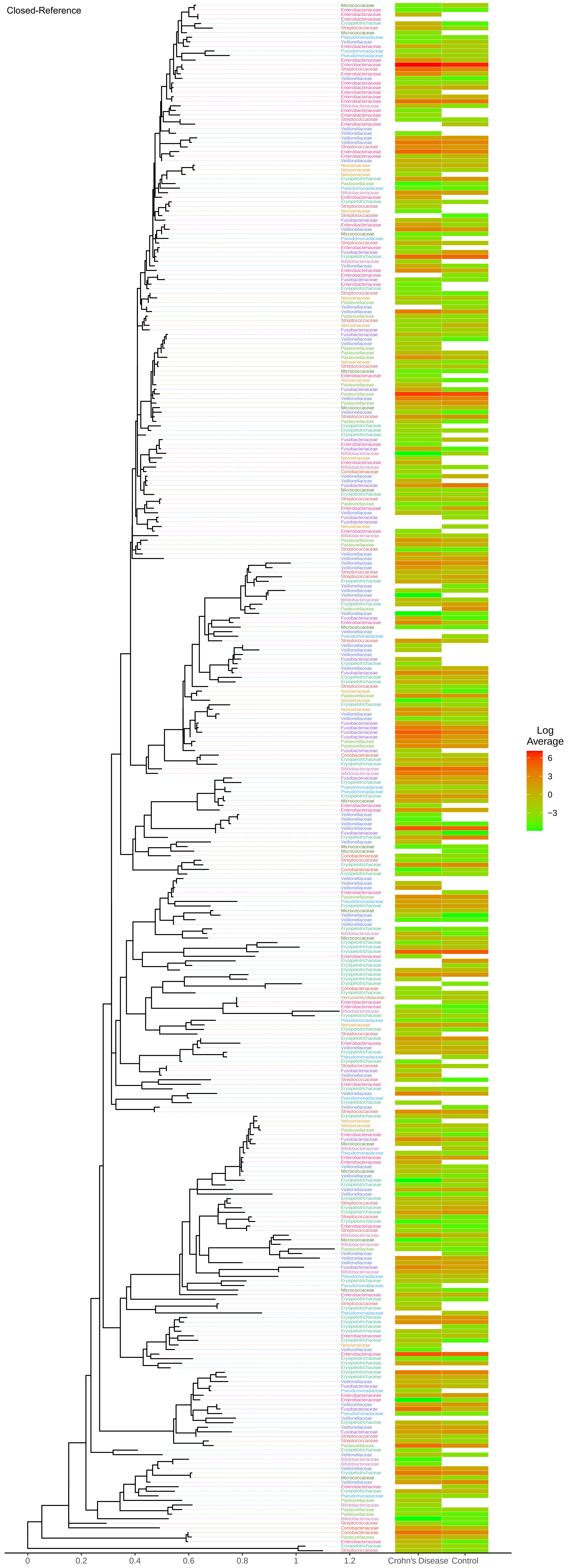
Closed-Reference
0
0.2
0.4
0.6
0.8
1
1.2
Crohn's Disease
Control
Log Average
6
3
0
−3

Figure S12. Phylogenetic tree from the closed-reference clustering method showing the mean log-transformed average count between the Crohn's Disease and Control on the Family level assignment.

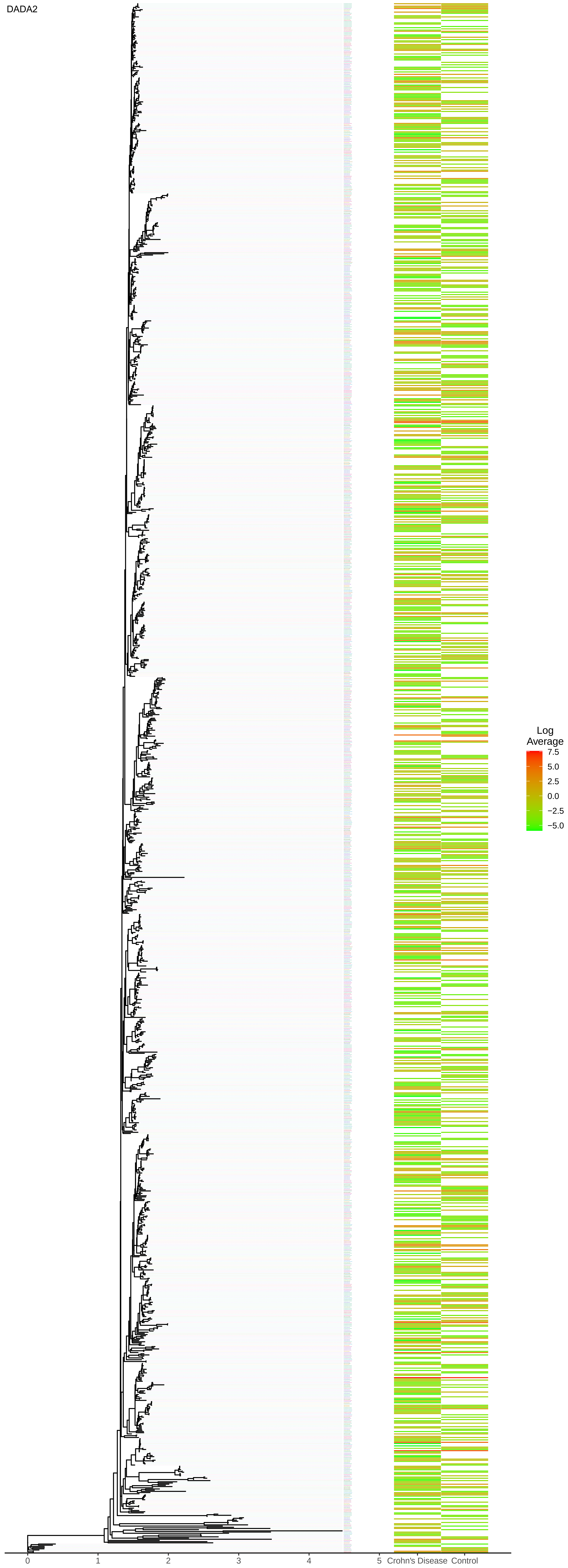
DADA2
Log
Average
7.5
5.0
2.5
0.0
−2.5
−5.0
0
1
2
3
4
5
Crohn's Disease
Control

Figure S13. Phylogenetic tree from the DADA2 clustering method showing the mean log-transformed average count between the Crohn's Disease and Control on the Family level assignment.

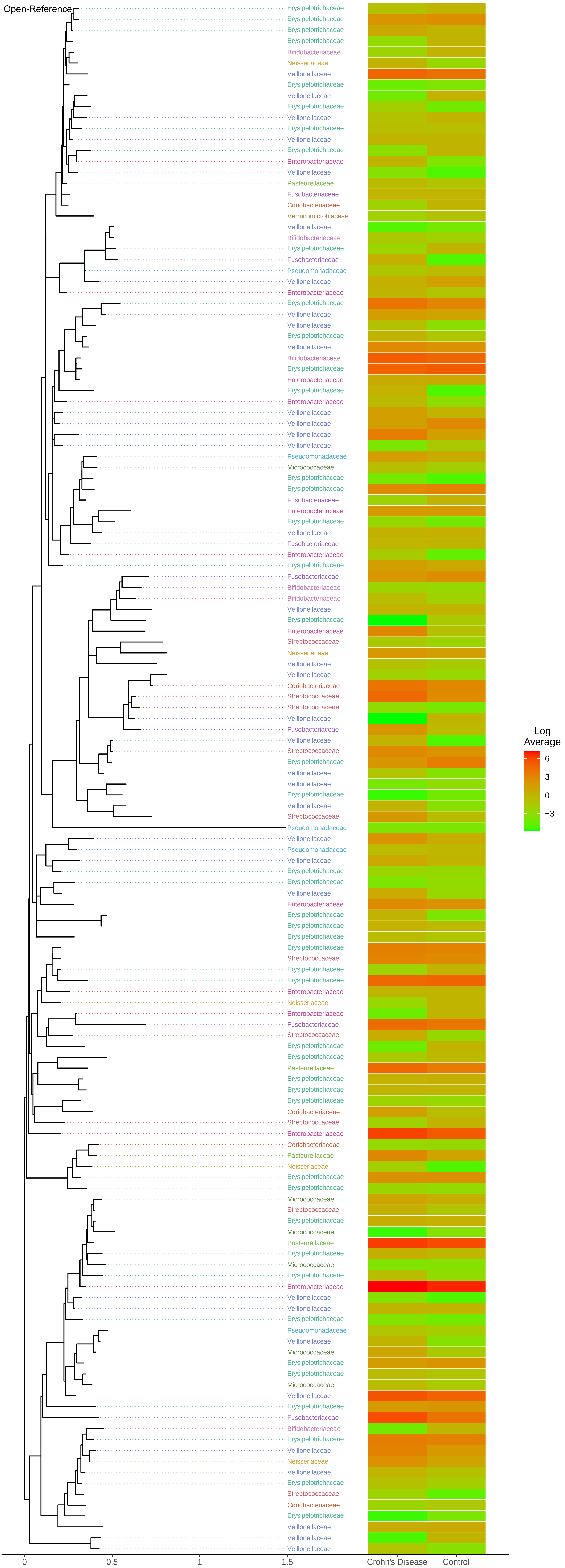

Open-Reference
Erysipelotrichaceae
Erysipelotrichaceae
Erysipelotrichaceae
Erysipelotrichaceae
Bifidobacteriaceae
Neisseriaceae
Veillonellaceae
Erysipelotrichaceae
Veillonellaceae
Erysipelotrichaceae
Veillonellaceae
Erysipelotrichaceae
Veillonellaceae
Erysipelotrichaceae
Enterobacteriaceae
Veillonellaceae
Pasteurellaceae
Fusobacteriaceae
Coriobacteriaceae
Verrucomicrobiaceae
Veillonellaceae
Bifidobacteriaceae
Erysipelotrichaceae
Fusobacteriaceae
Pseudomonadaceae
Veillonellaceae
Enterobacteriaceae
Erysipelotrichaceae
Veillonellaceae
Veillonellaceae
Erysipelotrichaceae
Veillonellaceae
Bifidobacteriaceae
Erysipelotrichaceae
Enterobacteriaceae
Erysipelotrichaceae
Enterobacteriaceae
Veillonellaceae
Veillonellaceae
Veillonellaceae
Veillonellaceae
Pseudomonadaceae
Micrococcaceae
Erysipelotrichaceae
Erysipelotrichaceae
Fusobacteriaceae
Enterobacteriaceae
Erysipelotrichaceae
Veillonellaceae
Fusobacteriaceae
Enterobacteriaceae
Erysipelotrichaceae
Fusobacteriaceae
Bifidobacteriaceae
Bifidobacteriaceae
Veillonellaceae
Erysipelotrichaceae
Enterobacteriaceae
Streptococcaceae
Neisseriaceae
Veillonellaceae
Veillonellaceae
Coriobacteriaceae
Streptococcaceae
Streptococcaceae
Veillonellaceae
Fusobacteriaceae
Veillonellaceae
Streptococcaceae
Erysipelotrichaceae
Veillonellaceae
Veillonellaceae
Erysipelotrichaceae
Veillonellaceae
Streptococcaceae
Pseudomonadaceae
Veillonellaceae
Pseudomonadaceae
Veillonellaceae
Erysipelotrichaceae
Erysipelotrichaceae
Veillonellaceae
Enterobacteriaceae
Erysipelotrichaceae
Erysipelotrichaceae
Erysipelotrichaceae
Erysipelotrichaceae
Streptococcaceae
Erysipelotrichaceae
Erysipelotrichaceae
Enterobacteriaceae
Neisseriaceae
Enterobacteriaceae
Fusobacteriaceae
Streptococcaceae
Erysipelotrichaceae
Erysipelotrichaceae
Pasteurellaceae
Erysipelotrichaceae
Erysipelotrichaceae
Erysipelotrichaceae
Coriobacteriaceae
Streptococcaceae
Enterobacteriaceae
Coriobacteriaceae
Pasteurellaceae
Neisseriaceae
Erysipelotrichaceae
Erysipelotrichaceae
Micrococcaceae
Streptococcaceae
Erysipelotrichaceae
Micrococcaceae
Pasteurellaceae
Erysipelotrichaceae
Micrococcaceae
Erysipelotrichaceae
Enterobacteriaceae
Veillonellaceae
Veillonellaceae
Erysipelotrichaceae
Pseudomonadaceae
Veillonellaceae
Micrococcaceae
Erysipelotrichaceae
Erysipelotrichaceae
Micrococcaceae
Veillonellaceae
Erysipelotrichaceae
Fusobacteriaceae
Bifidobacteriaceae
Erysipelotrichaceae
Veillonellaceae
Neisseriaceae
Veillonellaceae
Erysipelotrichaceae
Streptococcaceae
Coriobacteriaceae
Erysipelotrichaceae
Veillonellaceae
Veillonellaceae
Veillonellaceae
0
0.5
1
1.5
Crohn's Disease
Control
Log
Average
6
3
0
−3

Figure S14. Phylogenetic tree from the open-reference clustering method showing the mean log-transformed average count between the Crohn's Disease and Control on the Family level assignment.

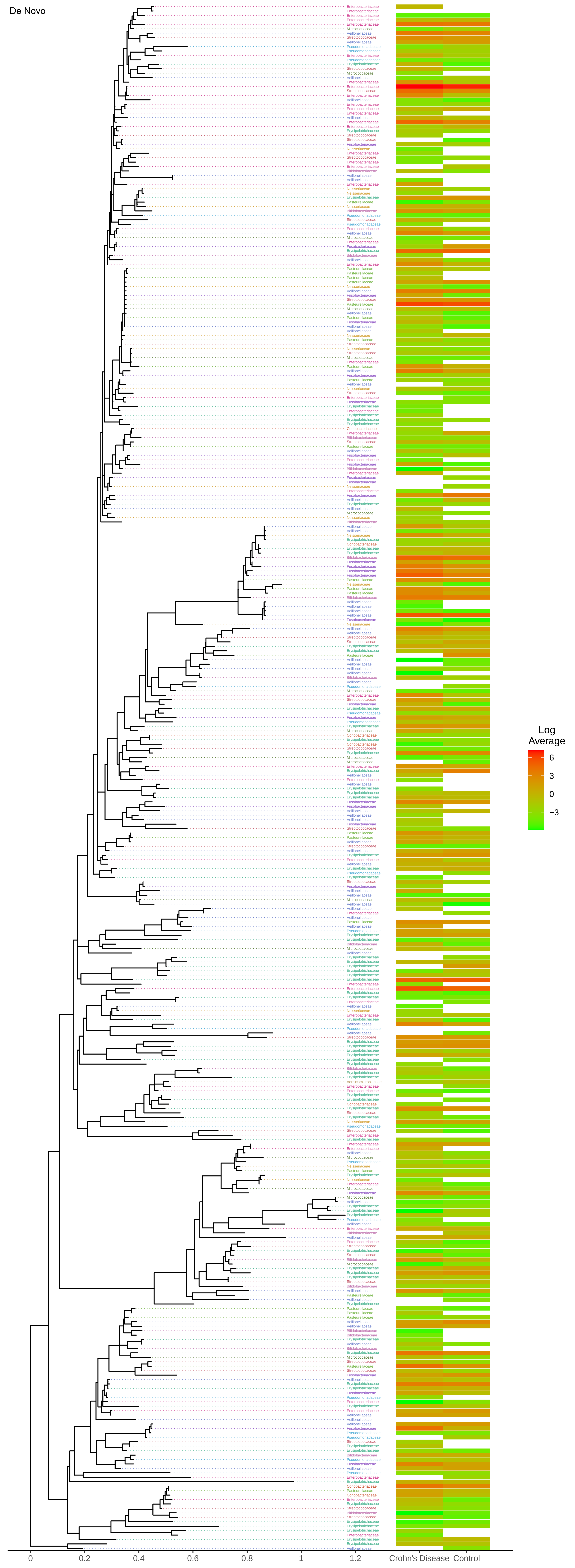
De Novo
Log Average
6
3
0
−3
Crohn's Disease
Control
0
0.2
0.4
0.6
0.8
1
1.2

Figure S15. Phylogenetic tree from the *de novo* clustering method showing the mean log-transformed average count between the Crohn's Disease and Control on the Family level assignment.
